# DNA methylation of *ARHGAP30* is negatively associated with *ARHGAP30* expression in lung adenocarcinoma, which reduces tumor immunity and is detrimental to patient survival

**DOI:** 10.18632/aging.203762

**Published:** 2021-12-15

**Authors:** Sheng Hu, Wenxiong Zhang, Jiayue Ye, Yang Zhang, Deyuan Zhang, Jinhua Peng, Dongliang Yu, Jianjun Xu, Yiping Wei

**Affiliations:** 1 Department of Thoracic Surgery, The Second Affiliated Hospital of Nanchang University, Nanchang, China

**Keywords:** ARHGAP30, lung adenocarcinoma, DNA methylation, gene set enrichment analysis (GSEA), tumor immunity

## Abstract

Rho-GTPase activating protein 30 (*ARHGAP30*) can enhance the intrinsic hydrolysis of GTP and regulates Rho-GTPase negatively. The relationship between *ARHGAP30* expression and lung adenocarcinoma is unclear. Therefore, the present study aimed to assess the differences in expression of *ARHGAP30* between lung adenocarcinoma tissues and normal tissues and the relationship between DNA methylation and *ARHGAP30* expression in lung adenocarcinoma. To determine the role of *ARHGAP30* expression in the prognosis and survival of patients with lung adenocarcinoma, gene set enrichment analysis of *ARHGAP30* was performed, comprising analyses of Kyoto Encyclopedia of Genes and Genomes pathways, Panther pathways, Reactome pathways, Wikipathways, Gene Ontology, Kinase Target Network, Transcription Factor Network, and a protein-protein interaction network. The association of *ARHGAP30* expression with tumor-infiltrating lymphocytes, immunostimulators, major histocompatibility complex molecules, chemokines, and chemokine receptors in lung adenocarcinoma tissues was also analyzed. DNA methylation of *ARHGAP30* correlated negatively with *ARHGAP30* expression. Patients with lung adenocarcinoma with high DNA methylation of *ARHGAP30* had poor prognosis. The prognosis of patients with lung adenocarcinoma with low *ARHGAP30* expression was also poor. *ARHGAP30* expression in lung adenocarcinoma correlated positively, whereas methylation of *ARHGAP30* correlated negatively, with levels of tumor infiltrating lymphocytes. Gene set enrichment analysis revealed that many pathways associated with *ARHGAP30* should be studied to improve the diagnosis, treatment, and prognosis of lung adenocarcinoma. We speculated that DNA methylation of *ARHGAP30* suppresses *ARHGAP30* expression, which reduces tumor immunity, leading to poor prognosis for patients with lung adenocarcinoma.

## INTRODUCTION

Worldwide, lung cancer cases and deaths are increasing. In 2018, GLOBOCAN [1] estimated that there were 2.09 million new cases (11.6% of the total number of cancer cases) and 1.76 million deaths (18.4% of the total number of cancer deaths), which is higher than the rate reported in 2012 (1.8 million new cases and 1.6 million deaths), making it the most common cause of cancer and cancer deaths in both men and women [2]. Lung cancer includes multiple subtypes, and the proportion of lung adenocarcinoma (LUAD) has increased in recent years. Despite significant advances in chemotherapy and molecular targeted therapy, the survival rate of LUAD remains unsatisfactory. Tumor recurrence and metastasis are major challenges in the clinical treatment of LUAD [3]. To improve the prognosis of patients with LUAD, more targeted molecules should be identified to diagnose, treat, and determine the prognosis of patients. We suggest that *ARHGAP30* might have potential as a new targeting molecule.

The Rho protein family belongs to the small GTP-binding proteins of the Ras superfamily (including the Ras, Rho, Rab, Ran, and Rrf families), which have a molecular weight between 20 and 30 kDa and control numerous signal transduction pathways as molecular switches in eukaryotic cells [4]. Rho proteins act as signal converters in the signal transduction pathway of cells, acting on the cytoskeleton or target proteins, and produce a variety of biological effects [5]. Rho GTPase activating protein 30 (*ARHGAP30*), a Rho-specific Rho GAP, has been reported to enhance the intrinsic hydrolysis of GTP and might regulate Rho GTPase negatively [6].

Recent studies have demonstrated a close relationship between Rho-GTPases and the development and metastasis of various human tumors [7]. In some studies on the relationship between *ARHGAP30* and cancer, upregulation of *ARHGAP30* attenuated pancreatic cancer progression by inactivating the β-catenin pathway [8]. In addition, *ARHGAP30* promotes p53 acetylation and function in colorectal cancer [9]. However, whether there is a difference in the expression of *ARHGAP30* in LUAD, a relationship between the expression of *ARHGAP30* in LUAD and DNA methylation, and whether these affect patient’s prognosis, survival, and tumor immune infiltration, are unclear and require further study.

This present study aimed to investigate the differential expression of *ARHGAP30* between LUAD tissues and normal tissues and the relationship between *ARHGAP30* expression and DNA methylation in LUAD. The role of *ARHGAP30* expression in the prognosis and survival of patients with LUAD was studied. In addition, gene set enrichment analysis (GSEA) of *ARHGAP30* was performed using various bioinformatic analyses, including Kyoto Encyclopedia of Genes and Genomes (KEGG) pathways, Panther pathways, Reactome pathways, Wikipathways, Gene ontology (GO; biological process, cellular component, and molecular function), Kinase Target Network, Transcription Factor Network, and a protein-protein interaction (PPI) network in the Biological General Repository for Interaction Datasets (BI-OGRID). The association of *ARHGAP30* expression with tumor-infiltrating lymphocytes (TILs), immunostimulators, major histocompatibility complex (MHC) molecules, chemokines, and chemokine receptors in LUAD tissues were also analyzed. We believe that *ARHGAP30* can be developed as a new biomarker for LUAD. The study of *ARHGAP30*-associated immune infiltration will provide a new direction for immunotherapy of lung adenocarcinoma.

## RESULTS

### Differential expression of the *ARHGAP30* mRNA and protein in LUAD tissues and normal tissues

**Figure 1:**
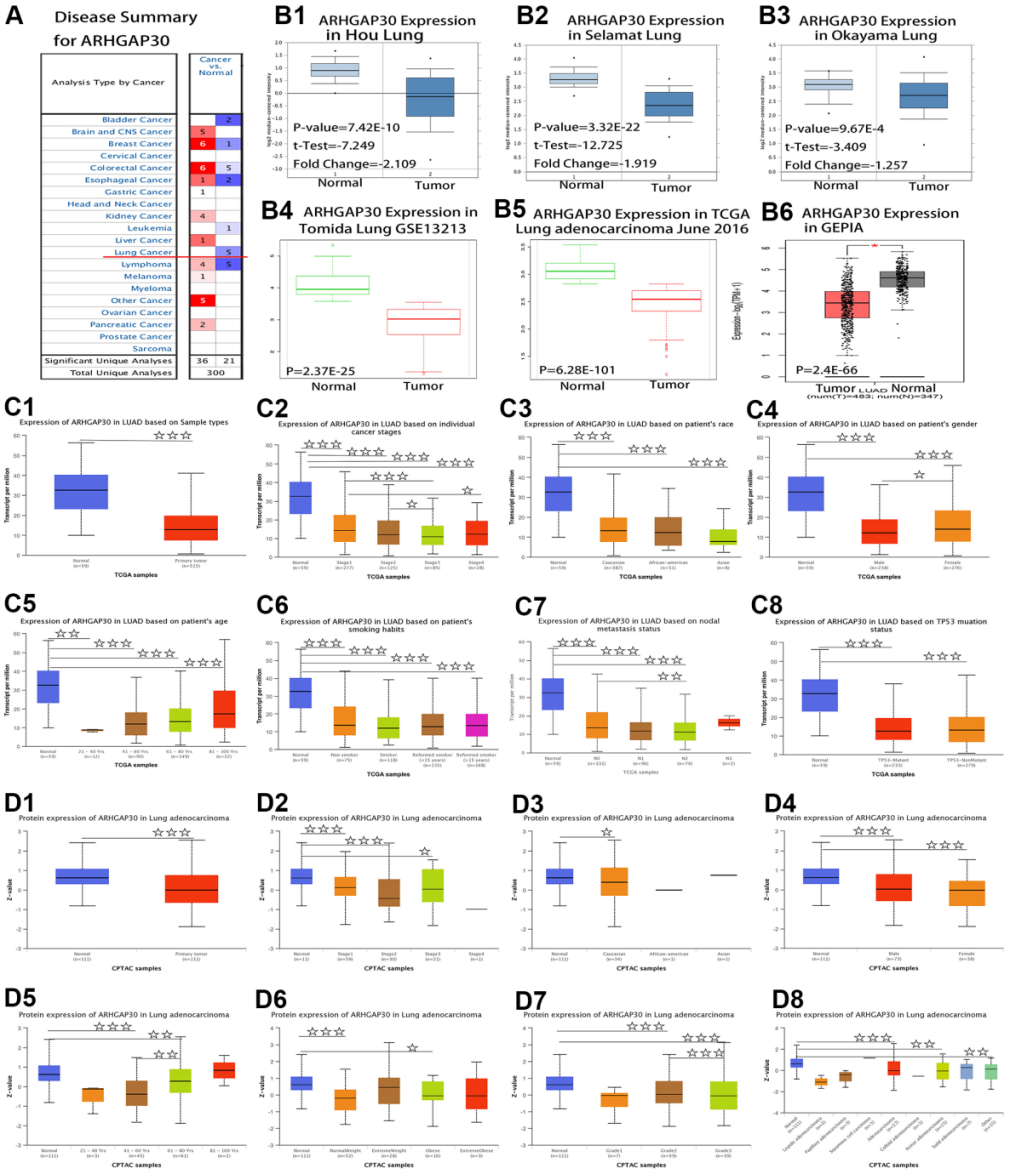
**Comparison of mRNA and protein expression of *ARHGAP30* in lung cancer tissues and normal tissues.** (**A**) Summary view of *ARHGAP30*. The transcription level of *ARHGAP30* in different types of cancer. P-value < 0.05, Note: The Z-score standardizes the color to describe the relative value in the row. Among them, red indicates overexpression or copy acquisition of genes in the analysis; blue indicates low expression or copy loss of genes in these analyses. Datasets comprised samples represented as microarray data measuring mRNA expression in primary tumors, cell lines, or xenografts. (**B**) Transcription of *ARHGAP30* in lung adenocarcinoma (from Oncomine, SurvExpress, and GEPIA databases). mRNA expression levels of *ARHGAP30* were significantly higher in lung adenocarcinoma than in normal tissue. (**B1**–**B3**) The fold change, associated p-values, and overexpression Gene Rank, based on Oncomine 4.5 analysis. Box plots show *ARHGAP30* mRNA levels in the Hou Lung, Selamat Lung, and Okayama Lung datasets. (**B4**, **B5**) The expression of *ARHGAP30* in LUAD based on SurvExpress analysis; (**B6**) The expression of *ARHGAP30* in LUAD based on GEPIA analysis; P values as described in the figure are statistically significant. (**C**) *ARHGAP30* transcription in subgroups of patients with lung adenocarcinoma, stratified based on sex, age, and other criteria (UALCAN). (**C1**) Sample types. (**C2**) Individual cancer stages. (**C3**) Ethnicity. (**C4**) Sex. (**C5**) Age. (**C6**) Smoking habits. (**C7**) Nodal metastasis status. (**C8**) *TP53* mutation status. ☆, P < 0.05; ☆☆, P < 0.01; ☆☆☆, P < 0.001. (**D**) Differential abundance of the *ARHGAP30* protein in patients with lung adenocarcinoma, stratified by sex, age, and other criteria. (**D1**) Sample types. (**D2**) Individual cancer stages. (**D3**) Ethnicity. (**D4**) Sex. (**D5**) Age. (**D6**) Weight. (**D7**) Tumor grade. (**D8**) Tumor histology. ☆, P < 0.05; ☆☆, P < 0.01; ☆☆☆, P < 0.001.

**Figure 2:**
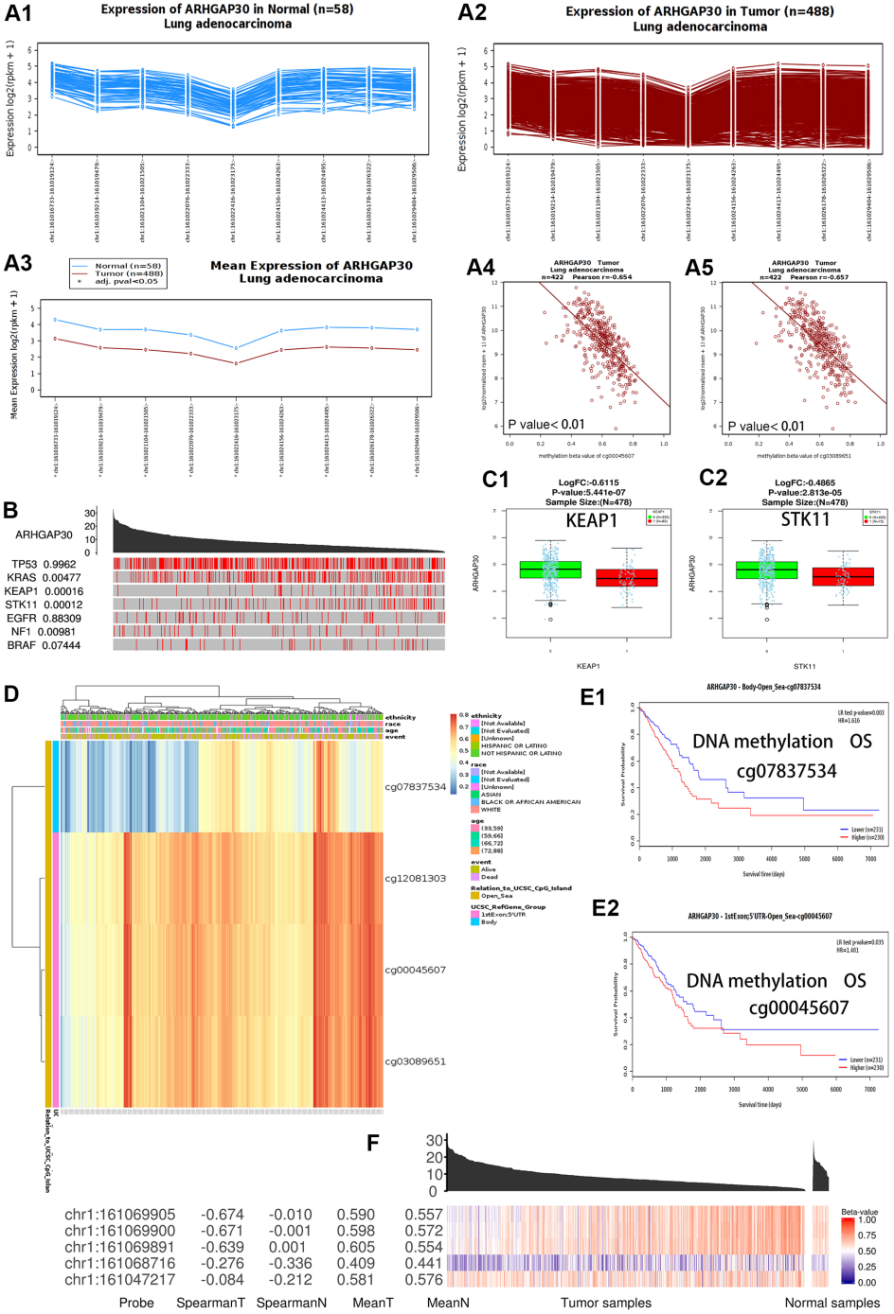
**DNA methylation and the differential expression of *ARHGAP30* between lung adenocarcinoma and normal tissues.** (**A**) The abundance of the different exons of the *ARHGAP30* gene shows an uneven balance in normal and tumor tissues in patients with lung adenocarcinoma according to the Wanderer database. (**A1**) Expression of *ARHGAP30* in normal tissues (n = 58); (**A2**) Expression of *ARHGAP30* in tumor tissues (n = 488); (**A3**) Comparison of the mean expression of *ARHGAP30* between normal tissue and lung adenocarcinoma tissue. (**A4**, **A5**) The expression of *ARHGAP30* correlated negatively with the level of DNA methylation. (**B**) Highly mutated genes and the expression of *ARHGAP30* in the TCGAportal database. The value adjacent to the highly mutated gene is the permutation test p-value of gene expression between the driver mutated (red) and not-mutated (gray) samples. (**C1**, **C2**) Box plots of the mRNA expression of *ARHGAP30* in lung adenocarcinoma before and after mutation of highly mutated genes (*KEAP1*, *STK11*) in the Linkedomics database. (**D**) Heat map of *ARHGAP30* methylation in lung adenocarcinoma. (**E1**, **E2**) Kaplan–Meier plots of the survival of patients with lung adenocarcinoma with different *ARHGAP30* DNA methylation levels (Different methylation probes cg07837534 and cg00045607 in the MethSurv database). (**F**) Gene expression and methylation of *ARHGAP30* in samples of primary tumors and solid tissues analyzed at the TCGAportal database. Spearman T: Spearman correlation between expression and methylation in primary tumor samples. Spearman N: Spearman correlation between expression and methylation in solid tissue standard samples. Mean T: Mean value of the methylation beta-value in primary tumor samples. Mean N: Mean value of methylation in normal solid tissue samples.

Figure 1A shows a summary view of the different transcriptional levels of *ARHGAP30* in various cancers in the Oncomine [10] database. The red line in the figure shows that the expression level of *ARHGAP30* in lung cancer tissue was significantly lower than that in normal tissue. Figure 1B1–1B6 show that the mRNA expression levels of *ARHGAP30* were considerably higher in LUAD than in normal tissue. Figure 1B1–1B3 show the fold change, associated p-values, and overexpression Gene Rank, based on Oncomine 4.5 analysis [10], including box plots of *ARHGAP30* mRNA levels in the Hou Lung, Selamat Lung, and Okayama Lung datasets. Figure 1B4, 1B5 show the expression of *ARHGAP30* in LUAD based on SurvExpress [11] analysis. Figure 1 (B6) shows the expression of *ARHGAP30* in LUAD based on GEPIA [12]. P values as described in the figure are statistically significant. According to analysis at the Warner [13] database, the abundance of the different exons of the *ARHGAP30* gene show an uneven balance between normal and tumor tissues in patients with LUAD (Figure 2A). Figure 2A1 shows the expression of *ARHGAP30* in normal tissues (n = 58) and Figure 2A2 shows the expression of *ARHGAP30* in tumor tissues (n = 488). The data shown in Figure 2A4, 2A5 indicates that *ARHGAP30* expression correlated negatively with the level of DNA methylation.

### Differential expression of ARHGAP30 mRNA in LUAD tissues and normal tissues

Figure 1C shows mRNA expression levels of *ARHGAP30* in subgroups of patients with LUAD, stratified based on sample type, individual cancer stage, ethnicity, sex, age, smoking habit, nodal metastasis status, and *TP53* mutation status (UALCAN [14]). The P-value of the comparison between each is shown in Supplementary Table 1. Figure 1C1 shows a significant difference between normal tissue and lung adenocarcinoma tissue (P < 0.001). Figure 1C2–1C8 show that in addition to the differential expression between tumor tissues and normal tissues, there were statistically significant differences between Stage 1 and Stage 3, Stage 1 and Stage 4, Stage 2 and Stage 3, male and female, and N0 and N2.

### Differential abundance of the ARHGAP30 protein in LUAD tissues and normal tissues

Figure 1D shows the protein levels of ARHGAP30 in subgroups of patients with LUAD, stratified based on sample type, individual cancer stage, ethnicity, sex, age, weight, tumor grade, and tumor histology (assessed using UALCAN [14] and CPTAC [15]). The P-value of the comparison between each is shown in Supplementary Table 2. Figure 1D1 shows a significant difference between normal tissue and LUAD tissue (P < 0.001). Figure 1D1–1D8 show that in addition to the differential abundance between tumor tissues and normal tissues, there were statistically significant differences between age 41–60 years and 61–80 years; and Grade 2 and Grade 3.

### Effect of mutations in common hypermutated genes and DNA methylation of *ARHGAP30* on the expression of *ARHGAP30* in lung adenocarcinoma versus normal tissues

The location of *ARHGAP30* methylation in the lung adenocarcinoma cases was on chromosome 1, 161015000 to 161,069905. Figure 2B shows that *ARHGAP30* expression was affected by some highly mutated genes in the analysis using the TCGAportal [16] database. Among them, *KRAS* (encoding KRAS proto-oncogene, GTPase), *KEAP1* (encoding kelch like ECH associated protein 1), *STK11* (encoding serine/threonine kinase 11), and *NF1* (encoding neurofibromin 1) genes had statistically significant P values. Figure 2C1, 2C2 show that *ARHGAP30* mRNA expression in LUAD was significantly lower than that in normal tissues after mutation of highly mutated genes (*KEAP1* and *STK11*) in the Linkedomics [17] database. These results indicate that mutations in *KEAP1* and *STK11* significantly reduce *ARHGAP30* gene expression and affect LUAD development.

Figure 2D shows a heatmap of *ARHGAP30* DNA methylation (using four probes: cg07837534, cg12081303, cg00045607, cg03089651) in LUAD based on analysis at the Methsurv [18] database, which showed that *ARHGAP30* DNA methylation levels were markedly increased in LUAD. A Kaplan–Meier map for patients with LUAD with different levels of *ARHGAP30* DNA methylation showed that patients with hypomethylation had a statistically significant better survival prognosis (Figure 2E1, 2E2) [18]. The Spearman correlation between expression and methylation in primary tumor samples was significantly higher than the Spearman correlation between expression and methylation in normal samples of solid tissues (Figure 2F) [16].

### Prediction of the prognosis of patients with LUAD according to *ARHGAP30* mRNA levels

**Figure 3:**
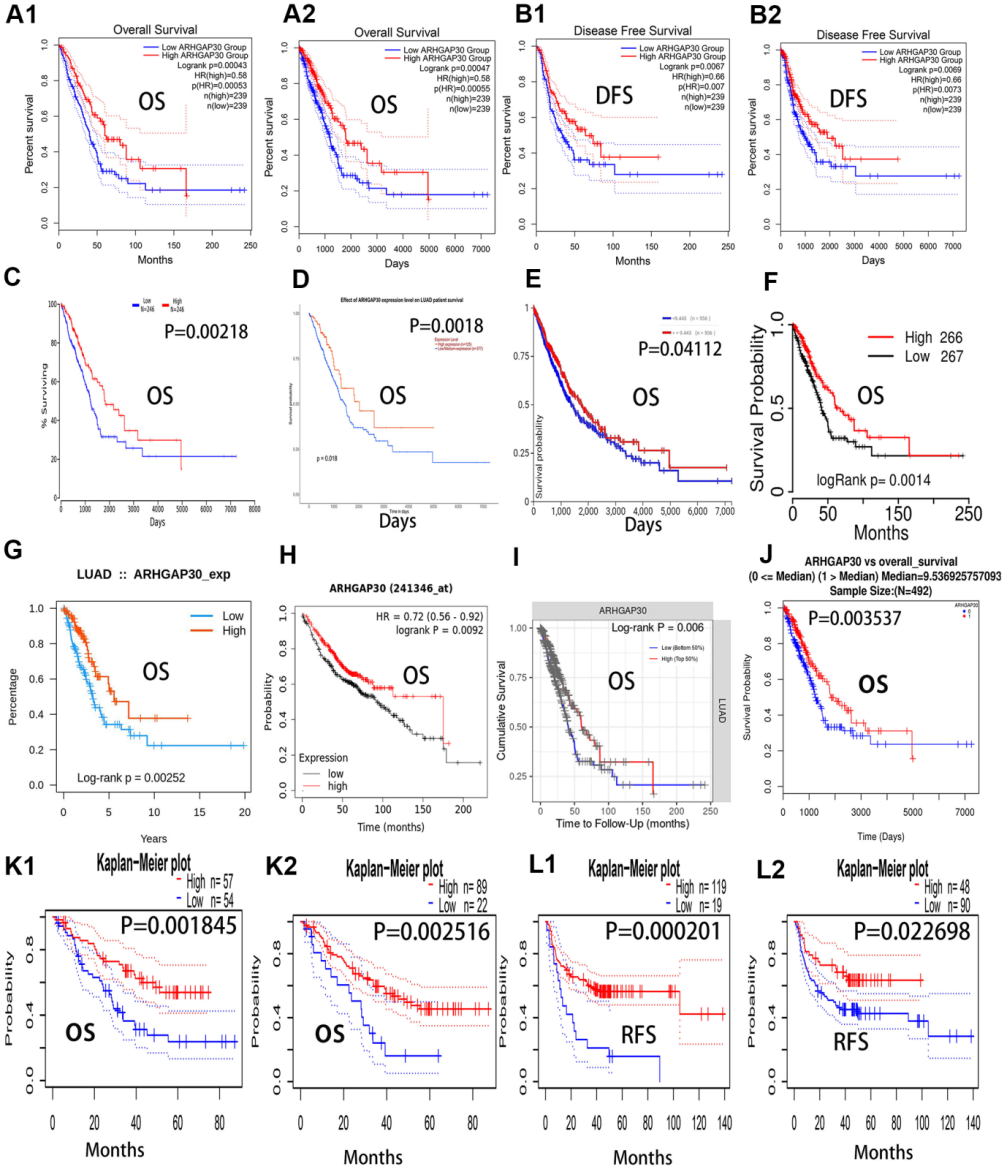
**Overall survival curves, recurrence-free survival curves, and disease-free survival curves of *ARHGAP30* in lung adenocarcinoma.** The blue curves represent patients with lung adenocarcinoma with low *ARHGAP30* expression, and the red curves represent patients with lung adenocarcinoma with high *ARHGAP30* expression. (**A1**, **A2**) Two overall survival curves (in months and days, respectively) from the GEPIA database; (**B1**, **B2**) Two disease-free survival (DFS) curves for *ARHGAP30* in the GEPIA database (in months and days, respectively). (**C**–**J**) Eight overall survival curves from the databases of Oncolnc, Ualcan, UCSC, TCGAportal, TISIDB, KMplot, TIMER, and Linkedomics, respectively. (**K1**, **K2**) Two survival curves representing the overall survival curves from the PrognoScan database. (**L1**, **L2**) Two survival curves representing recurrence-free survival curves from the PrognoScan database.

We found that the prognosis of patients with LUAD with high *ARHGAP30* mRNA expression levels was significantly better than that of patients with low *ARHGAP30* mRNA expression levels, as demonstrated by the 12 overall survival curves shown in Figure 3 (all P < 0.01). Figure 3A1, 3A2 represent the two overall survival curves from the GEPIA [12] database; Figure 3C–3J represent the eight overall survival curves from the Oncolnc [19], Ualcan [14], UCSC [20], TCGA portal [16], TISIDB [21], KMplot [22], TIMER [23], and Linkedomics [17] databases. The two survival curves in Figure 3K1, 3K2 represent the overall survival curves from the PrognoScan [24] database. Figure 3B1, 3B2 show two disease-free survival curves from the GEPIA database, which indicate that the prognosis of patients with LUAD with high expression of *ARHGAP30* mRNA was significantly higher than that of patients with low expression of *ARHGAP30* mRNA (P < 0.01). The two survival curves in Figure 3L1, 3L2 represent recurrence-free survival curves from the PrognoScan [24] database), which show that the prognosis of patients with LUAD with high expression of *ARHGAP30* mRNA were significantly higher than that of patients with low expression of *ARHGAP30* mRNA (P < 0.05).

### Genes, miRNAs, and lncRNAs correlated highly with *ARHGAP30* in lung adenocarcinoma

**Figure 4:**
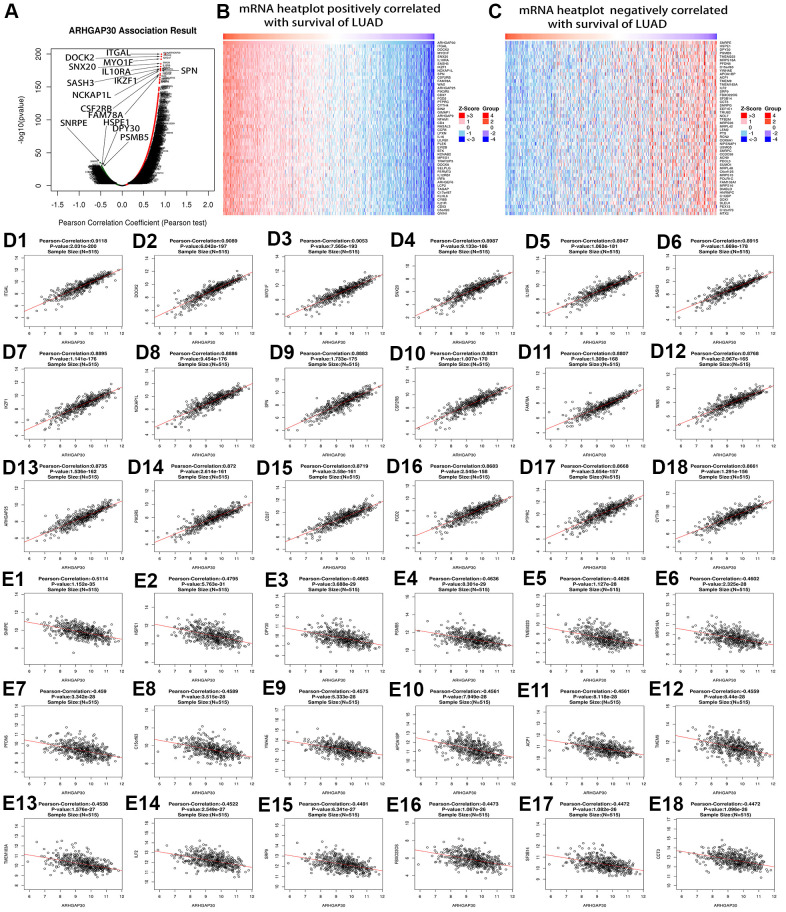
**Genes that correlated highly with *ARHGAP30* in lung adenocarcinoma (LUAD).** (**A**) Volcano map of *ARHGAP30*-correlated genes in LUAD, the red dots on the right represent the positively related genes, and the green dots on the left represent the negatively related genes. (**B**, **C**) Heat maps showing the genes that correlated positively and negatively with *ARHGAP30* in LUAD (top 50). Red indicates positively correlated genes; green indicates negatively correlated genes. (**D1**–**D18**) Scatter plots of the first 18 genes that correlated positively with *ARHGAP30* in LUAD. (**E1**–**E18**) Scatter plots of the first 18 genes that correlated negatively with *ARHGAP30* in LUAD.

We analyzed the genes and microRNAs (miRNAs) that correlated with *ARHGAP30* based on the Linkedomics [17] database. Figure 4A shows a volcano plot of genes that correlated highly with *ARHGAP30* in LUAD. Figure 4B shows a heatmap of genes that correlated highly and positively with *ARHGAP30* in LUAD. Figure 4C shows a heatmap of genes that correlated highly and negatively with *ARHGAP30* in LUAD. Figure 4D1–4D18 show scatter plots of the top 18 genes that correlated positively with *ARHGAP30* in LUAD: *ITGAL*, *DOCK2*, *MYO1F*, *SNX20*, *IL10RA*, *SASH3*, *IKZF1*, *NCKAP1L*, *SPN*, *CSF2RB*, *FAM78A*, *WAS*, *ARHGAP25*, *PIK3R5*, *CD37*, *FGD2*, *PTPRC*, and *CYTH4*. Figure 4E1–4E18 show scatter plots of the top 18 genes that correlated negatively with *ARHGAP30* in LUAD: *SNRPE*, *HSPE1*, *DPY30*, *PSMB5*, *TMEM223*, *MRPS18A*, *PFDN6*, *C15orf63*, *YWHAE*, *APOA1BP*, *ACP1*, *TMEM9*, *TMEM183A*, *ILF2*, *SRP9*, *FBXO22OS*, *SF3B14*, and *CCT3*.

**Figure 5:**
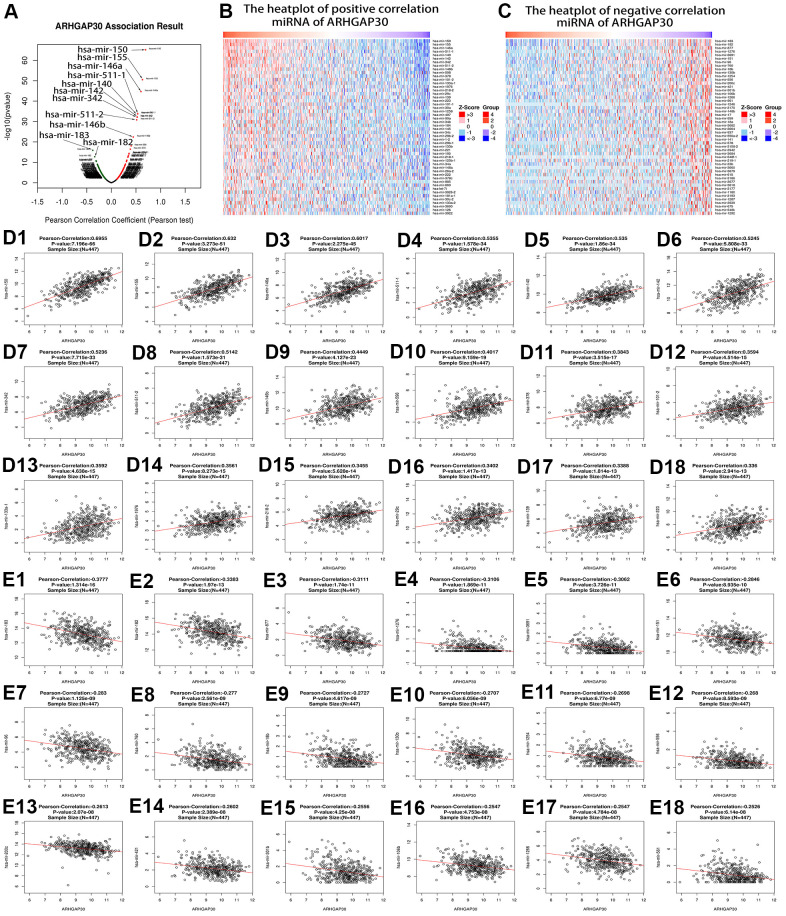
**MiRNAs correlated highly with *ARHGAP30* in lung adenocarcinoma (LUAD).** (**A**) Volcano map of *ARHGAP30*-correlated miRNAs in LUAD, the red dots on the right represent the positively associated miRNAs, and the green dots on the left represent the negatively associated miRNAs. (**B**, **C**) Heat maps showing the miRNAs that correlated positively and negatively with *ARHGAP30* in LUAD (top 50). Red indicates positively correlated miRNAs; green indicates negatively correlated miRNAs. (**D1**–**D18**) Scatter plots of the first 18 miRNAs that correlated positively with *ARHGAP30* in LUAD. (**E1**–**E18**) Scatter plots of the first 18 miRNAs that correlated negatively with *ARHGAP30* in LUAD.

Figure 5A shows a volcano plot of miRNAs that correlated highly with *ARHGAP30* in LUAD. Figure 5B shows a heatmap of miRNAs that correlated highly and positively with *ARHGAP30* in LUAD. Figure 5C shows a heatmap of miRNAs that correlated highly and negatively with *ARHGAP30* in LUAD. Figure 5D1–5D18 show scatter plots of the top 18 miRNAs that correlated positively with *ARHGAP30* in LUAD: hsa-mir-150, hsa-mir-155, hsa-mir-146a, hsa-mir-511-1, hsa-mir-140, hsa-mir-142, hsa-mir-342, hsa-mir-511-2, hsa-mir-146b, hsa-mir-598, hsa-mir-378, hsa-mir-101-2, hsa-mir-133a-1, hsa-mir-1976, hsa-mir-218-2, hsa-mir-29c, hsa-mir-139, and hsa-mir-223. Figure 5E1–5E18 show scatter plots of the top 18 mRNAs that corelated negatively with *ARHGAP30* in LUAD: hsa-mir-183, hsa-mir-182, hsa-mir-877, hsa-mir-1276, hsa-mir-3691, hsa-mir-151, hsa-mir-96, hsa-mir-760, hsa-mir-18b, hsa-mir-130b, hsa-mir-1254, hsa-mir-556, hsa-mir-200c, hsa-mir-421, hsa-mir-301b, hsa-mir-106b, hsa-mir-1266 and hsa-mir-561.

**Figure 6:**
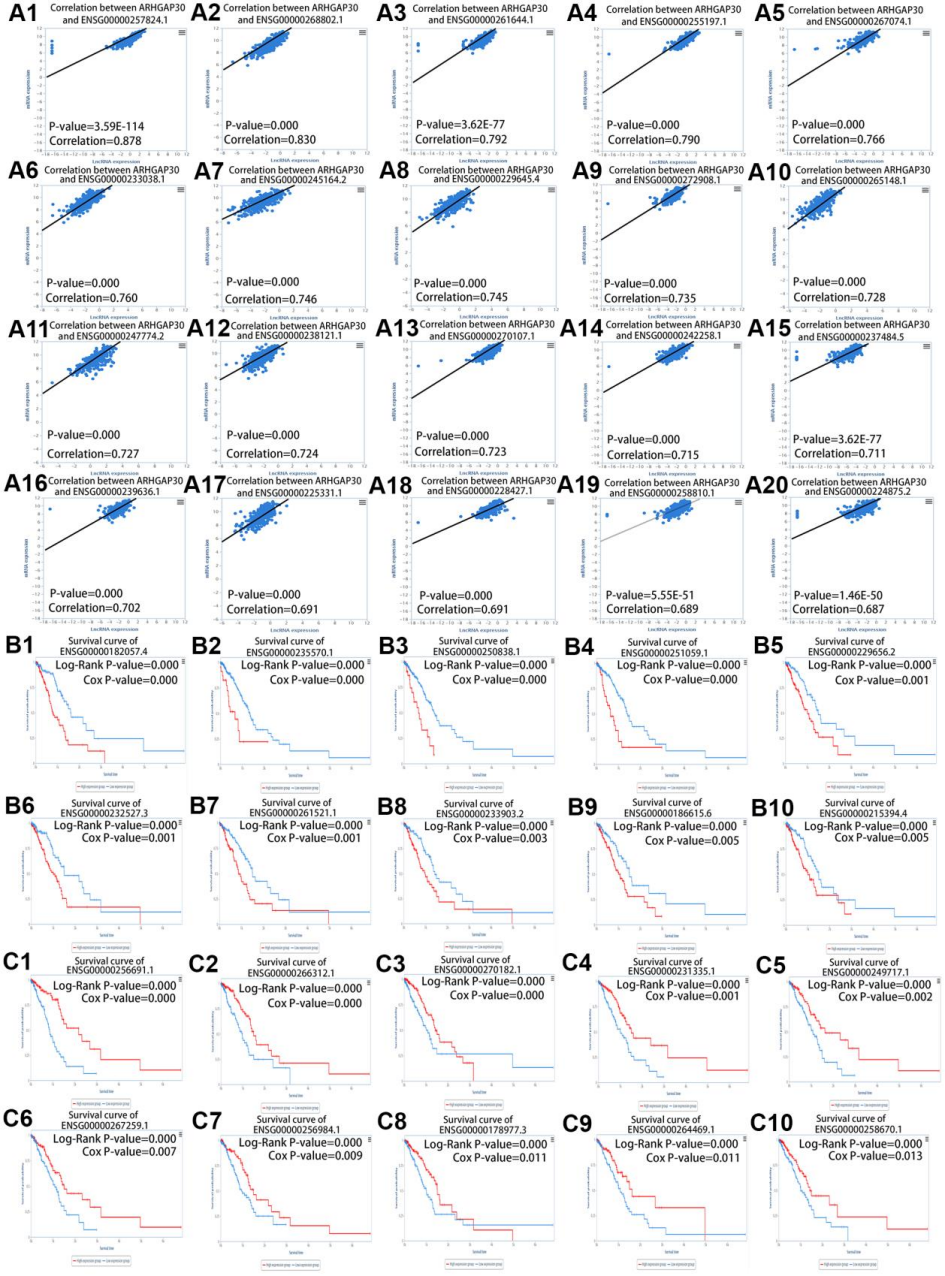
**LncRNAs correlated highly with *ARHGAP30* in lung adenocarcinoma (LUAD).** (**A1**–**A20**) Scatter plots of lncRNAs that are positively associated with *ARHGAP30* in LUAD. (**B1**–**B10**) *ARHGAP30* correlated lncRNAs, in which low expression has a better prognosis according to the survival curve of the lncRNAs. (**C1**–**C10**) *ARHGAP30* correlated lncRNAs, in which high expression has a better prognosis according to the survival curve of lncRNAs.

We analyzed the long noncoding RNAs (lncRNAs) that correlated with *ARHGAP30* based on the TANRIC [25] database. Figure 6A1–6A20 show scatter plots of lncRNAs that are highly and positively correlated with *ARHGAP30* in LUAD: ENSG00000257824.1, ENSG00000268802.1, ENSG00000261644.1, ENSG00000255197.1, ENSG00000267074.1, ENSG00000233038.1, ENSG00000245164.2, ENSG00000229645.4, ENSG00000272908.1, ENSG00000265148.1, ENSG00000247774.2, ENSG00000238121.1, ENSG00000270107.1, ENSG00000242258.1, ENSG00000237484.5, ENSG00000239636.1, ENSG00000225331.1, ENSG00000228427.1, ENSG00000258810.1, ENSG00000224875.2. Figure 6B1–6B10 show survival curves with a better prognosis for those lncRNAs with low expression associated with *ARHGAP30*: ENSG00000182057.4, ENSG00000235570.1, ENSG00000250838.1, ENSG00000251059.1, ENSG00000229656.2, ENSG00000232527.3, ENSG00000261521.1, ENSG00000233903.2, ENSG00000186615.6, and ENSG00000215394.4 (all P < 0.05). Figure 6C1–6C10 show survival curves with a better prognosis for highly expressed lncRNAs associated with *ARHGAP30*: ENSG00000256691.1, ENSG00000266312.1, ENSG00000270182.1, ENSG00000231335.1, ENSG00000249717.1, ENSG00000267259.1, ENSG00000256984.1, ENSG00000178977.3, ENSG00000264469.1, and ENSG00000258670.1 (all P < 0.05).

### Gene set enrichment analysis of *ARHGAP30* in lung adenocarcinoma

We performed gene set enrichment analysis (GSEA) [26] of *ARHGAP30* using the Linkedomics [17] database for KEGG Pathway [27], Panther Pathway [28], Reactome Pathway [29], Wikipathway [30], Gene ontology Biological Process [31, 32], Gene ontology Cellular Component [31, 32], Gene ontology Molecular Function [31, 32], Kinase Target Network, Transcription Factor Network, and PPI BIOGRID Network [33]. We identified many genes related to tumor immunity in the enrichment results.

**Figure 7:**
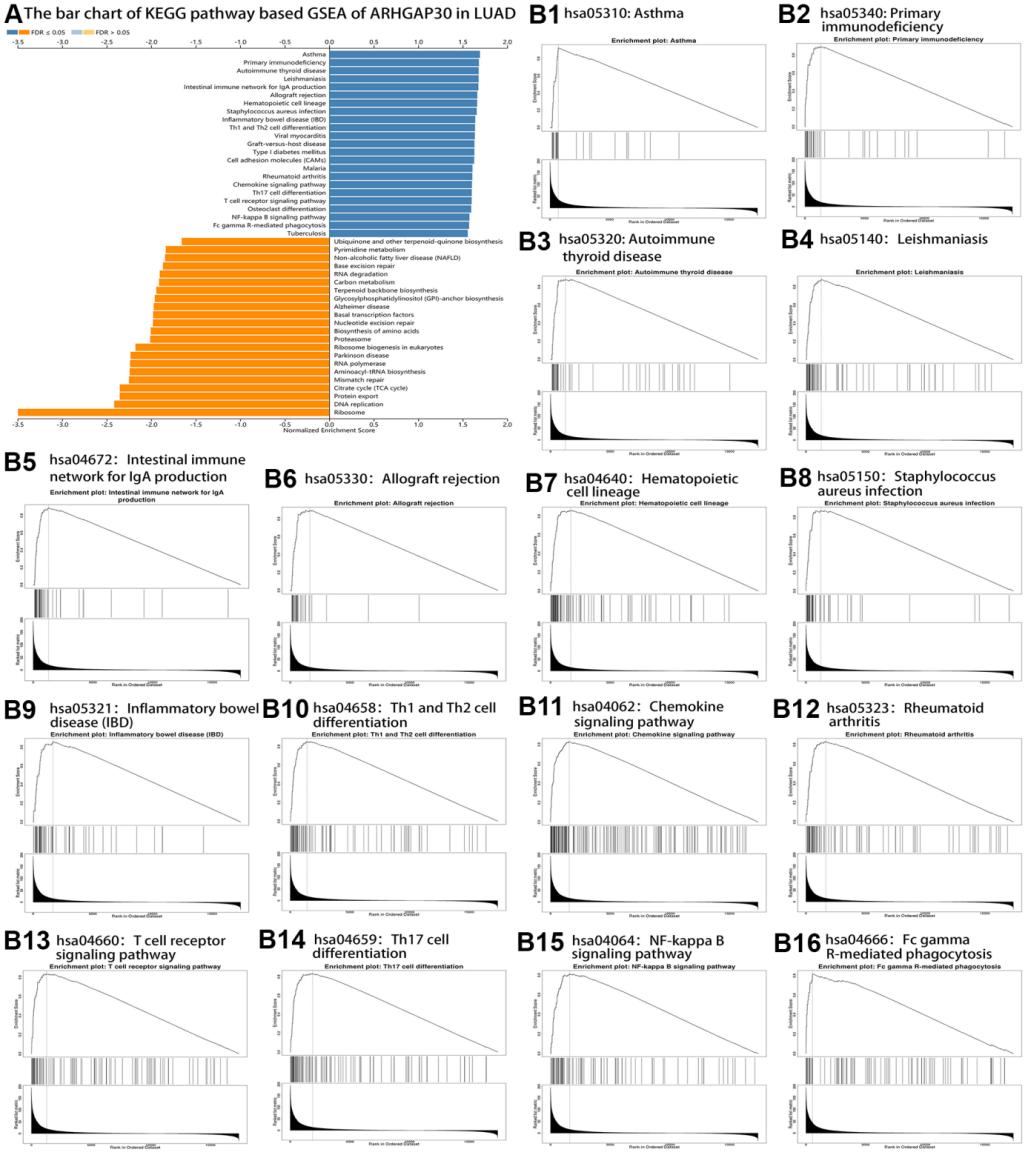
**KEGG pathway-based GSEA of *ARHGAP30* in lung adenocarcinoma (LUAD).** (**A**) Bar chart of KEGG pathway-based GSEA of *ARHGAP30* in LUAD (FDR < 0.05). (**B1**–**B16**) GSEA enrichment analysis Plots of 16 tumor immune-related KEGG gene sets (FDR < 0.05).

The results of KEGG pathway enrichment analysis are shown in Figure 7A. Significantly enriched pathways were identified using false discovery rate (FDR) less than 0.05 and the absolute value of the normalized enrichment score greater than 1. Figure 7B1, 7B2 show the enrichment profiles of some statistically significant gene sets in the KEGG analysis. Supplementary Figures 1–9 show the bar charts and enrichment profiles for *ARHGAP30* GSEA of the Panther Pathway, Reactome Pathway, Wikipathway, Gene ontology Biological Process, Gene ontology Cellular Component, Gene ontology Molecular Function, Kinase Target Network, Transcription Factor Network, and PPI BIOGRID Network. Tables 1–10 detail the results of *ARHGAP30* GSEA for the Panther Pathway, Reactome Pathway, Wikipathway, Gene ontology Biological Process, Gene ontology Cellular Component, Gene ontology Molecular Function, Kinase Target Network, Transcription Factor Network, and PPI BIOGRID Network, respectively, which were statistically significant (absolute normalized enrichment score (NES values greater than 1, FDR and P values less than 0.05).

**Table 1:** KEGG pathway based gene set enrichment analysis of *ARHGAP30* in lung adenocarcinoma.

**Gene set**	**Description**	**Size**	**Leading edge number**	**ES**	**NES**	**P Value**	**FDR**
hsa05310	Asthma	28	18	0.92455	1.6939	0	0
hsa05340	Primary immunodeficiency	36	24	0.89598	1.6822	0	0
hsa05320	Autoimmune thyroid disease	48	27	0.88334	1.6799	0	0
hsa05140	Leishmaniasis	71	41	0.88206	1.6794	0	0
hsa04672	Intestinal immune network for IgA production	45	34	0.88949	1.676	0	0
hsa05330	Allograft rejection	35	31	0.89287	1.6643	0	0
hsa04640	Hematopoietic cell lineage	93	58	0.86219	1.6628	0	0
hsa05150	Staphylococcus aureus infection	52	36	0.87392	1.6576	0	0
hsa05321	Inflammatory bowel disease (IBD)	63	41	0.85768	1.6417	0	0
hsa04658	Th1 and Th2 cell differentiation	90	46	0.85202	1.6391	0	0
hsa05416	Viral myocarditis	56	32	0.8628	1.6368	0	0
hsa05332	Graft-versus-host disease	37	27	0.86783	1.6327	0	0
hsa04940	Type I diabetes mellitus	41	30	0.86296	1.6313	0	0
hsa04514	Cell adhesion molecules (CAMs)	137	54	0.8432	1.6295	0	0
hsa05012	Parkinson disease	115	66	-0.59262	-2.2319	0	0
hsa03020	RNA polymerase	31	21	-0.74745	-2.237	0	0
hsa00970	Aminoacyl-tRNA biosynthesis	43	30	-0.67799	-2.2413	0	0
hsa03430	Mismatch repair	23	11	-0.80357	-2.2495	0	0
hsa00020	Citrate cycle (TCA cycle)	30	19	-0.7776	-2.3534	0	0
hsa03060	Protein export	22	17	-0.79558	-2.3539	0	0
hsa03030	DNA replication	36	19	-0.76463	-2.4152	0	0
hsa03010	Ribosome	131	100	-0.83153	-3.4961	0	0
hsa04062	Chemokine signaling pathway	185	73	0.82904	1.6039	0	9.82E-05
hsa05323	Rheumatoid arthritis	85	42	0.83117	1.6084	0	0.000104
hsa05144	Malaria	46	27	0.84172	1.6104	0	0.000111
hsa04660	T cell receptor signaling pathway	98	35	0.83662	1.6002	0	0.000176
hsa04659	Th17 cell differentiation	105	54	0.83277	1.6019	0	0.000185
hsa04380	Osteoclast differentiation	126	57	0.83591	1.5957	0	0.00025
hsa04064	NF-kappa B signaling pathway	90	42	0.81436	1.5757	0	0.00091
hsa04666	Fc gamma R-mediated phagocytosis	86	17	0.81881	1.5724	0	0.001016
hsa03008	Ribosome biogenesis in eukaryotes	70	37	-0.65008	-2.1763	0	0.001061
hsa05152	Tuberculosis	174	65	0.80199	1.5575	0	0.001535
hsa00900	Terpenoid backbone biosynthesis	22	17	-0.74335	-1.9428	0	0.002387
hsa03420	Nucleotide excision repair	45	16	-0.59231	-1.9806	0	0.002387
hsa00563	Glycosylphosphatidylinositol (GPI)-anchor biosynthesis	25	10	-0.71989	-1.9597	0	0.002546
hsa01230	Biosynthesis of amino acids	69	27	-0.59257	-2.0052	0	0.002604
hsa05010	Alzheimer disease	152	67	-0.49713	-1.9717	0	0.002728
hsa01200	Carbon metabolism	110	38	-0.47465	-1.9118	0	0.002808
hsa03050	Proteasome	44	34	-0.60203	-2.0108	0	0.002865
hsa03022	Basal transcription factors	44	18	-0.63104	-1.9781	0	0.002938
hsa03018	RNA degradation	73	27	-0.50119	-1.9041	0	0.003183
hsa03410	Base excision repair	33	13	-0.68657	-1.8682	0	0.004523
hsa04932	Non-alcoholic fatty liver disease (NAFLD)	143	55	-0.46729	-1.8446	0	0.005729
hsa00240	Pyrimidine metabolism	96	42	-0.4934	-1.836	0	0.005911
hsa00130	Ubiquinone and other terpenoid-quinone biosynthesis	11	5	-0.76111	-1.6567	0	0.027882

**Table 2:** Panther pathway gene set enrichment analysis of *ARHGAP30* in lung adenocarcinoma.

**Gene set**	**Description**	**Size**	**Leading edge number**	**ES**	**NES**	**P Value**	**FDR**
P00053	T cell activation	75	30	0.87572	1.6754	0	0
P02738	De novo purine biosynthesis	26	16	-0.79062	-2.2412	0	0
P00017	DNA replication	19	10	-0.79041	-2.2625	0	0
P00023	General transcription regulation	28	14	-0.72986	-2.101	0	0.001287
P00010	B cell activation	58	19	0.84216	1.5819	0	0.004295
P00055	Transcription regulation by bZIP transcription factor	45	14	-0.58101	-1.8961	0	0.005792
P00038	JAK/STAT signaling pathway	15	9	0.9035	1.5543	0.002381	0.006872
P02746	Heme biosynthesis	12	6	-0.73501	-1.7337	0.011364	0.013998
P02740	De novo pyrimidine ribonucleotides biosynthesis	10	7	-0.79533	-1.7549	0.009901	0.014894
P00031	Inflammation mediated by chemokine and cytokine signaling pathway	196	72	0.78311	1.524	0	0.015463
P00051	TCA cycle	10	5	-0.83656	-1.7588	0	0.017377
P02739	De novo pyrimidine deoxyribonucleotide biosynthesis	13	8	-0.74772	-1.7772	0	0.019307
P00009	Axon guidance mediated by netrin	30	12	0.81439	1.4941	0.008511	0.035736
P00014	Cholesterol biosynthesis	12	8	-0.76183	-1.6443	0.010101	0.039902

**Table 3:** Wikipathway gene set enrichment analysis of *ARHGAP30* in lung adenocarcinoma.

**Gene set**	**Description**	**Size**	**Leading edge number**	**ES**	**NES**	**P Value**	**FDR**
R-HSA-110373	Resolution of AP sites via the multiple-nucleotide patch replacement pathway	26	15	-0.80592	-2.1643	0	0
R-HSA-114604	GPVI-mediated activation cascade	34	14	0.86846	1.613	0	0.003124
R-HSA-1268020	Mitochondrial protein import	52	35	-0.82458	-2.784	0	0
R-HSA-1461973	Defensins	21	5	0.92843	1.7135	0	0
R-HSA-162599	Late Phase of HIV Life Cycle	121	59	-0.61857	-2.4395	0	0
R-HSA-191859	snRNP Assembly	49	19	-0.78096	-2.5186	0	0
R-HSA-194441	Metabolism of non-coding RNA	49	19	-0.78096	-2.5186	0	0
R-HSA-198933	Immunoregulatory interactions between a Lymphoid and a non-Lymphoid cell	122	79	0.86427	1.6654	0	0.000368
R-HSA-202427	Phosphorylation of CD3 and TCR zeta chains	20	20	0.93356	1.6673	0.002353	0.00042
R-HSA-202430	Translocation of ZAP-70 to Immunological synapse	17	16	0.94274	1.6844	0	0
R-HSA-202433	Generation of second messenger molecules	30	22	0.94177	1.7411	0	0
R-HSA-2029482	Regulation of actin dynamics for phagocytic cup formation	60	14	0.83348	1.5954	0	0.005648
R-HSA-2172127	DAP12 interactions	38	21	0.87591	1.6582	0	0.000327
R-HSA-2299718	Condensation of Prophase Chromosomes	69	47	-0.66539	-2.1895	0	0
R-HSA-2424491	DAP12 signaling	29	15	0.88744	1.6332	0	0.00084
R-HSA-379724	tRNA Aminoacylation	42	32	-0.71306	-2.3694	0	0
R-HSA-380108	Chemokine receptors bind chemokines	45	26	0.84855	1.5991	0	0.004982
R-HSA-388841	Costimulation by the CD28 family	67	34	0.88459	1.7064	0	0
R-HSA-389948	PD-1 signaling	21	20	0.93832	1.7049	0	0
R-HSA-451927	Interleukin-2 family signaling	44	28	0.89201	1.6924	0	0
R-HSA-512988	Interleukin-3, Interleukin-5 and GM-CSF signaling	47	24	0.86993	1.6512	0	0.000294
R-HSA-5621480	Dectin-2 family	24	10	0.90122	1.6503	0	0.000245
R-HSA-5668599	RHO GTPases Activate NADPH Oxidases	13	5	0.94977	1.6075	0	0.003718
R-HSA-5696399	Global Genome Nucleotide Excision Repair (GG-NER)	84	31	-0.63145	-2.2073	0	0
R-HSA-606279	Deposition of new CENPA-containing nucleosomes at the centromere	63	36	-0.70907	-2.5507	0	0
R-HSA-6781827	Transcription-Coupled Nucleotide Excision Repair (TC-NER)	77	34	-0.6929	-2.399	0	0
R-HSA-6782135	Dual incision in TC-NER	64	27	-0.72714	-2.3136	0	0
R-HSA-6783783	Interleukin-10 signaling	45	28	0.86974	1.6503	0	0.000267
R-HSA-6790901	rRNA modification in the nucleus and cytosol	52	35	-0.80009	-2.6392	0	0
R-HSA-69202	Cyclin E associated events during G1/S transition	82	50	-0.61312	-2.2508	0	0
R-HSA-69206	G1/S Transition	124	75	-0.64433	-2.4821	0	0
R-HSA-69618	Mitotic Spindle Checkpoint	101	56	-0.67804	-2.3397	0	0
R-HSA-69656	Cyclin A:Cdk2-associated events at S phase entry	84	50	-0.60739	-2.4501	0	0
R-HSA-72165	mRNA Splicing - Minor Pathway	46	20	-0.74059	-2.3252	0	0
R-HSA-73863	RNA Polymerase I Transcription Termination	30	12	-0.81293	-2.5196	0	0
R-HSA-73864	RNA Polymerase I Transcription	106	43	-0.61126	-2.3211	0	0
R-HSA-73884	Base Excision Repair	39	17	-0.77946	-2.3177	0	0
R-HSA-73933	Resolution of Abasic Sites (AP sites)	39	17	-0.77946	-2.3177	0	0
R-HSA-774815	Nucleosome assembly	63	36	-0.70907	-2.5507	0	0
R-HSA-877300	Interferon gamma signaling	90	53	0.82409	1.5933	0	0.00553
R-HSA-912526	Interleukin receptor SHC signaling	27	15	0.88441	1.6345	0	0.000905
R-HSA-983695	Antigen activates B Cell Receptor (BCR) leading to generation of second messengers	32	18	0.86	1.6179	0	0.002744

**Table 4:** Reactome pathway gene set enrichment analysis of *ARHGAP30* in lung adenocarcinoma.

**Gene set**	**Description**	**Size**	**Leading edge number**	**ES**	**NES**	**P Value**	**FDR**
WP3937	Microglia Pathogen Phagocytosis Pathway	40	25	0.93221	1.7523	0	0
WP69	T-Cell antigen Receptor (TCR) Signaling Pathway	89	39	0.86566	1.6825	0	0
WP3863	T-Cell antigen Receptor (TCR) pathway during Staphylococcus aureus infection	61	26	0.86662	1.6615	0	0
WP3945	TYROBP Causal Network	59	40	0.88146	1.6593	0	0
WP2328	Allograft Rejection	87	55	0.86119	1.6499	0	0
WP286	IL-3 Signaling Pathway	48	22	0.87343	1.6334	0	0
WP78	TCA Cycle (aka Krebs or citric acid cycle)	18	13	-0.79775	-2.1053	0	0
WP4752	Base Excision Repair	31	13	-0.76263	-2.224	0	0
WP4521	Glycosylation and related congenital defects	25	13	-0.78449	-2.2261	0	0
WP466	DNA Replication	36	19	-0.75101	-2.3665	0	0
WP623	Oxidative phosphorylation	37	27	-0.81707	-2.3904	0	0
WP405	Eukaryotic Transcription Initiation	42	24	-0.77435	-2.4676	0	0
WP477	Cytoplasmic Ribosomal Proteins	88	72	-0.77946	-2.4707	0	0
WP107	Translation Factors	50	28	-0.76662	-2.4884	0	0
WP4324	Mitochondrial complex I assembly model OXPHOS system	44	39	-0.84395	-2.6711	0	0
WP111	Electron Transport Chain (OXPHOS system in mitochondria)	73	61	-0.83256	-2.9456	0	0
WP4595	Urea cycle and associated pathways	21	9	-0.73691	-2.0795	0	0.000281
WP531	DNA Mismatch Repair	22	10	-0.77183	-2.0484	0	0.000515
WP619	Type II interferon signaling (IFNG)	37	22	0.87609	1.625	0	0.000533
WP4753	Nucleotide Excision Repair	44	16	-0.59965	-2.0373	0	0.000713
WP2446	Retinoblastoma Gene in Cancer	86	45	-0.55877	-1.9707	0	0.001443
WP4022	Pyrimidine metabolism	83	39	-0.49658	-1.9718	0	0.001546
WP4559	Interactions between immune cells and microRNAs in tumor microenvironment	28	20	0.86424	1.6013	0	0.001864
WP4585	Cancer immunotherapy by PD-1 blockade	23	15	0.88715	1.6016	0	0.00205
WP49	IL-2 Signaling Pathway	42	17	0.84445	1.6036	0	0.002278
WP22	IL-9 Signaling Pathway	17	9	0.92271	1.6042	0	0.00233
WP205	IL-7 Signaling Pathway	25	12	0.89998	1.5928	0	0.003417
WP4146	Macrophage markers	9	8	0.97473	1.5863	0	0.003594
WP3929	Chemokine signaling pathway	163	62	0.82524	1.5876	0	0.003728
WP4494	Selective expression of chemokine receptors during T-cell polarization	29	20	0.86987	1.5752	0	0.003837
WP581	EPO Receptor Signaling	26	8	0.87123	1.5768	0	0.003844
WP2849	Hematopoietic Stem Cell Differentiation	55	18	0.84073	1.5807	0	0.003852
WP4582	Cancer immunotherapy by CTLA4 blockade	14	7	0.91643	1.5725	0	0.004038
WP2583	T-Cell Receptor and Co-stimulatory Signaling	29	13	0.86168	1.5679	0	0.004807
WP23	B Cell Receptor Signaling Pathway	96	39	0.81089	1.5636	0	0.005498
WP453	Inflammatory Response Pathway	30	15	0.84311	1.5595	0	0.005676
WP24	Peptide GPCRs	73	19	0.81715	1.5604	0	0.005858
WP2453	TCA Cycle and Deficiency of Pyruvate Dehydrogenase complex	16	11	-0.77333	-1.9018	0	0.006183
WP127	IL-5 Signaling Pathway	40	13	0.82934	1.5565	0	0.006321
WP4553	FBXL10 enhancement of MAP/ERK signaling in diffuse large B-cell lymphoma	32	10	-0.59305	-1.8368	0	0.011093
WP1946	Cori Cycle	17	8	-0.72333	-1.8214	0	0.012022
WP4629	Computational Model of Aerobic Glycolysis	11	7	-0.77655	-1.8124	0	0.013017
WP197	Cholesterol Biosynthesis Pathway	13	9	-0.76865	-1.7715	0.009901	0.019786
WP4240	Regulation of sister chromatid separation at the metaphase-anaphase transition	15	9	-0.68148	-1.7149	0	0.035479
WP438	Non-homologous end joining	10	2	-0.78427	-1.6835	0.024194	0.040727
WP4320	The effect of progerin on the involved genes in Hutchinson-Gilford Progeria Syndrome	36	14	-0.57494	-1.6836	0	0.042578

**Table 5:** Gene ontology biological process based gene set enrichment analysis of *ARHGAP30* in lung adenocarcinoma.

**Gene set**	**Description**	**Size**	**Leading edge number**	**ES**	**NES**	**P Value**	**FDR**
GO:0006968	cellular defense response	53	26	0.85607	1.6667	0	0
GO:0000959	mitochondrial RNA metabolic process	33	22	-0.67592	-2.0538	0	0
GO:0002181	cytoplasmic translation	84	50	-0.58607	-2.0597	0	0
GO:0098781	ncRNA transcription	93	46	-0.54515	-2.0641	0	0
GO:0071806	protein transmembrane transport	59	27	-0.70316	-2.1031	0	0
GO:0034502	protein localization to chromosome	68	39	-0.61386	-2.1257	0	0
GO:0042769	DNA damage response, detection of DNA damage	38	15	-0.70411	-2.1428	0	0
GO:0006490	oligosaccharide-lipid intermediate biosynthetic process	20	9	-0.8074	-2.1678	0	0
GO:0006354	DNA-templated transcription, elongation	84	27	-0.54275	-2.1898	0	0
GO:0045454	cell redox homeostasis	59	24	-0.65482	-2.1915	0	0
GO:0061641	CENP-A containing chromatin organization	24	16	-0.77476	-2.2312	0	0
GO:0036260	RNA capping	30	13	-0.79033	-2.3135	0	0
GO:0006353	DNA-templated transcription, termination	69	26	-0.69744	-2.3511	0	0
GO:0072350	tricarboxylic acid metabolic process	38	21	-0.73574	-2.4276	0	0
GO:0033108	mitochondrial respiratory chain complex assembly	68	53	-0.82238	-2.4489	0	0
GO:0010257	NADH dehydrogenase complex assembly	49	41	-0.83836	-2.4807	0	0
GO:0006289	nucleotide-excision repair	106	39	-0.64825	-2.4996	0	0
GO:0006414	translational elongation	123	82	-0.83503	-3.2155	0	0
GO:0032623	interleukin-2 production	63	31	0.83578	1.6105	0	0.000291
GO:0032609	interferon-gamma production	102	56	0.84241	1.6107	0	0.000317
GO:0070661	leukocyte proliferation	272	122	0.84138	1.6349	0	0.000349
GO:0002285	lymphocyte activation involved in immune response	165	68	0.83527	1.6137	0	0.000349
GO:0007159	leukocyte cell-cell adhesion	310	135	0.83054	1.6142	0	0.000388
GO:0001773	myeloid dendritic cell activation	27	15	0.86561	1.6095	0	0.000403
GO:0050690	regulation of defense response to virus by virus	29	12	0.85941	1.639	0	0.000437
GO:0002250	adaptive immune response	366	175	0.835	1.6177	0	0.000437
GO:0042110	T cell activation	437	184	0.83599	1.6255	0	0.000499
GO:0050867	positive regulation of cell activation	298	126	0.82659	1.608	0	0.000499
GO:0032633	interleukin-4 production	34	21	0.88557	1.6508	0	0.000582
GO:0045730	respiratory burst	27	10	0.90536	1.6256	0	0.000582
GO:0031123	RNA 3'-end processing	111	48	-0.62236	-1.9837	0	0.000584
GO:0016073	snRNA metabolic process	82	42	-0.56867	-1.9865	0	0.000611
GO:0051131	chaperone-mediated protein complex assembly	19	6	-0.74976	-2.0021	0	0.00064
GO:0042107	cytokine metabolic process	106	43	0.83001	1.6024	0	0.000698
GO:0071706	tumor necrosis factor superfamily cytokine production	133	54	0.82167	1.6013	0	0.000764
GO:1990868	response to chemokine	86	44	0.84852	1.6524	0	0.000873
GO:0030101	natural killer cell activation	79	30	0.83376	1.5967	0	0.000873
GO:0002694	regulation of leukocyte activation	461	199	0.82149	1.5987	0	0.000924
GO:0042113	B cell activation	221	86	0.82221	1.5887	0	0.000998
GO:0050866	negative regulation of cell activation	172	78	0.82699	1.5914	0	0.001011
GO:0002764	immune response-regulating signaling pathway	452	159	0.80813	1.5818	0	0.001215
GO:0032613	interleukin-10 production	46	24	0.83341	1.5734	0	0.001293

**Table 6:** Gene ontology cellular component based gene set enrichment analysis of *ARHGAP30* in lung adenocarcinoma.

**Gene set**	**Description**	**Size**	**Leading edge number**	**ES**	**NES**	**P Value**	**FDR**
GO:0042611	MHC protein complex	19	16	0.91235	1.6397	0	0
GO:0036452	ESCRT complex	23	12	-0.7271	-1.9814	0	0
GO:0101031	chaperone complex	21	13	-0.7488	-2.089	0	0
GO:0005732	small nucleolar ribonucleoprotein complex	20	14	-0.84007	-2.2357	0	0
GO:0005844	polysome	70	44	-0.64071	-2.2843	0	0
GO:0009295	nucleoid	36	27	-0.76327	-2.3211	0	0
GO:1905368	peptidase complex	85	54	-0.68339	-2.4793	0	0
GO:0005681	spliceosomal complex	155	64	-0.60446	-2.5676	0	0
GO:0030964	NADH dehydrogenase complex	43	39	-0.82377	-2.6221	0	0
GO:0070069	cytochrome complex	29	22	-0.87423	-2.6756	0	0
GO:0070469	respiratory chain	84	62	-0.82349	-2.6858	0	0
GO:0120114	Sm-like protein family complex	69	28	-0.78085	-2.7326	0	0
GO:0030684	preribosome	66	39	-0.73361	-2.7355	0	0
GO:0001772	immunological synapse	32	17	0.85713	1.5928	0	0.000759
GO:1905348	endonuclease complex	23	10	-0.7109	-1.8954	0	0.003019
GO:0098552	side of membrane	459	171	0.80484	1.5734	0	0.00354
GO:0098636	protein complex involved in cell adhesion	35	14	0.83327	1.5509	0	0.00531
GO:0042629	mast cell granule	21	9	0.85342	1.5417	0	0.006069
GO:0001891	phagocytic cup	21	12	0.85394	1.536	0	0.006575
GO:0042581	specific granule	152	44	0.77662	1.5083	0	0.010431
GO:0070820	tertiary granule	155	43	0.77958	1.5136	0	0.010837
GO:0005657	replication fork	62	21	-0.52303	-1.7674	0	0.012616
GO:1990204	oxidoreductase complex	95	61	-0.47317	-1.7327	0	0.017008
GO:0031970	organelle envelope lumen	73	28	-0.44485	-1.7196	0	0.017172
GO:0030667	secretory granule membrane	279	76	0.75106	1.4744	0	0.023264
GO:0005697	telomerase holoenzyme complex	20	10	-0.62191	-1.6713	0.017241	0.032323
GO:0043235	receptor complex	391	143	0.73726	1.437	0	0.047337
GO:0036019	endolysosome	19	9	0.82188	1.4317	0.004587	0.047999

**Table 7:** Gene ontology molecular function-based gene set enrichment analysis of *ARHGAP30* in lung adenocarcinoma.

**Gene set**	**Size**	**Leading edge number**	**ES**	**NES**	**P Value**	**FDR**	**Description**
GO:0042287	MHC protein binding	24	16	0.90783	1.6451	0	0
GO:0008135	translation factor activity, RNA binding	81	34	-0.59488	-2.1067	0	0
GO:0043021	ribonucleoprotein complex binding	117	44	-0.55984	-2.1205	0	0
GO:0000049	tRNA binding	50	32	-0.61345	-2.1332	0	0
GO:0015002	heme-copper terminal oxidase activity	24	16	-0.84644	-2.3002	0	0
GO:0030515	snoRNA binding	28	19	-0.80939	-2.3053	0	0
GO:0016675	oxidoreductase activity, acting on a heme group of donors	25	16	-0.84613	-2.3757	0	0
GO:0019843	rRNA binding	60	42	-0.74059	-2.4081	0	0
GO:0051082	unfolded protein binding	108	52	-0.69233	-2.6499	0	0
GO:0003735	structural constituent of ribosome	154	119	-0.83969	-3.289	0	0
GO:0016502	nucleotide receptor activity	22	14	0.87811	1.6115	0	0.00054724
GO:0035586	purinergic receptor activity	26	16	0.86825	1.6126	0	0.00082086
GO:0004896	cytokine receptor activity	88	49	0.84639	1.6087	0	0.0016417
GO:0017069	snRNA binding	34	10	-0.67977	-1.9375	0	0.0022837
GO:0003684	damaged DNA binding	67	26	-0.49758	-1.9239	0	0.0028547
GO:0016779	nucleotidyltransferase activity	114	44	-0.47695	-1.9243	0	0.0031142
GO:0035004	phosphatidylinositol 3-kinase activity	81	25	0.82041	1.5905	0	0.0032834
GO:0019865	immunoglobulin binding	22	12	0.86362	1.5831	0.0022272	0.003518
GO:0038187	pattern recognition receptor activity	20	11	0.87926	1.5833	0	0.0041043
GO:0052813	phosphatidylinositol bisphosphate kinase activity	73	24	0.81306	1.5743	0	0.0045147
GO:0043548	phosphatidylinositol 3-kinase binding	30	11	0.84191	1.546	0	0.0073877
GO:0003823	antigen binding	52	25	0.83357	1.5482	0.0020367	0.0080262
GO:0019239	deaminase activity	27	9	0.84449	1.5368	0	0.010149
GO:0042169	SH2 domain binding	33	9	0.83581	1.5289	0	0.010229
GO:0015026	coreceptor activity	39	20	0.83108	1.5324	0	0.010261
GO:0019955	cytokine binding	119	53	0.7923	1.5183	0	0.012547
GO:1990782	protein tyrosine kinase binding	76	18	0.79568	1.5158	0	0.012587
GO:0031491	nucleosome binding	66	20	-0.49926	-1.7891	0	0.016689
GO:0017056	structural constituent of nuclear pore	22	3	-0.61094	-1.758	0	0.023653
GO:0016790	thiolester hydrolase activity	31	13	-0.5909	-1.7292	0	0.028166
GO:0038024	cargo receptor activity	77	26	0.76716	1.4694	0	0.03776
GO:0104005	hijacked molecular function	70	14	0.77566	1.4646	0	0.039884
GO:0004713	protein tyrosine kinase activity	174	56	0.75063	1.4588	0	0.042685
GO:0003697	single-stranded DNA binding	93	41	-0.46853	-1.6551	0	0.044247
GO:0051087	chaperone binding	96	27	-0.46803	-1.6357	0	0.045003
GO:0030506	ankyrin binding	20	2	0.81515	1.4498	0.0090703	0.04856
GO:0051540	metal cluster binding	59	26	-0.53488	-1.6196	0	0.048846

**Table 8:** Kinase target network gene set enrichment analysis of *ARHGAP30* in lung adenocarcinoma.

**Gene set**	**Description**	**Size**	**Leading edge number**	**ES**	**NES**	**P Value**	**FDR**
Kinase_LYN	LYN proto-oncogene, Src family tyrosine kinase	50	23	0.88163	1.69	0	0
Kinase_SYK	spleen associated tyrosine kinase	35	20	0.88807	1.6638	0	0
Kinase_LCK	LCK proto-oncogene, Src family tyrosine kinase	43	25	0.87754	1.6409	0	0
Kinase_HCK	HCK proto-oncogene, Src family tyrosine kinase	23	14	0.90568	1.6236	0	0.000453
Kinase_BTK	Bruton tyrosine kinase	9	4	0.96245	1.5569	0	0.014843
Kinase_FGR	FGR proto-oncogene, Src family tyrosine kinase	12	7	0.90291	1.5354	0.004819	0.023015
Kinase_FYN	FYN proto-oncogene, Src family tyrosine kinase	66	21	0.79674	1.5309	0	0.023306
Kinase_PRKCQ	protein kinase C theta	28	10	0.83313	1.5386	0.002179	0.023834
Kinase_ITK	IL2 inducible T-cell kinase	8	6	0.95805	1.5163	0	0.030592
Kinase_JAK3	Janus kinase 3	12	8	0.8914	1.5164	0.005051	0.033991

**Table 9:** Transcription factor network gene set enrichment analysis of *ARHGAP30* in lung adenocarcinoma.

**Gene set**	**Size**	**Leading edge number**	**ES**	**NES**	**P Value**	**FDR**
V$PU1_Q6	211	48	0.7456	1.4539	0	0.027156
V$PEA3_Q6	242	73	0.74837	1.4483	0	0.027497
RACCACAR_V$AML_Q6	241	66	0.74025	1.4434	0	0.028904
RGAGGAARY_V$PU1_Q6	460	107	0.7462	1.4553	0	0.030226
STTTCRNTTT_V$IRF_Q6	175	68	0.75524	1.4614	0	0.030856
V$PAX5_02	15	7	0.85689	1.4665	0.009346	0.035138
V$ISRE_01	234	77	0.75508	1.4722	0	0.038255
V$IRF_Q6	229	78	0.76436	1.482	0	0.039042
V$ELF1_Q6	220	69	0.77176	1.5138	0	0.039672
V$ETS_Q4	238	55	0.72616	1.4108	0	0.043159
TGTYNNNNNRGCARM_UNKNOWN	81	26	0.73366	1.4113	0	0.046284
V$ICSBP_Q6	230	75	0.71252	1.3885	0	0.047526
V$ETS1_B	237	76	0.71314	1.3914	0	0.047543
V$STAT6_02	241	60	0.71256	1.3852	0	0.0477
V$AML_Q6	239	75	0.72796	1.4135	0	0.047915
GGGNNTTTCC_V$NFKB_Q6_01	130	51	0.76183	1.4879	0	0.048173
YNTTTNNNANGCARM_UNKNOWN	66	16	0.73294	1.3927	0.00202	0.048562

**Table 10:** PPI BIOGRID network gene set enrichment analysis of *ARHGAP30* in lung adenocarcinoma.

**Gene set**	**Size**	**Leading edge number**	**ES**	**NES**	**P Value**	**FDR**
PPI_BIOGRID_M856	27	20	-0.80351	-2.2385	0	0
PPI_BIOGRID_M299	43	23	-0.77865	-2.3323	0	0
PPI_BIOGRID_M422	41	25	-0.78055	-2.38	0	0
PPI_BIOGRID_M298	50	37	-0.8034	-2.6225	0	0
PPI_BIOGRID_M300	49	42	-0.88664	-3.0801	0	0
PPI_BIOGRID_M272	85	44	-0.53652	-2.1103	0	0.000404
PPI_BIOGRID_M428	43	23	-0.62913	-2.1148	0	0.000471
PPI_BIOGRID_M441	36	15	-0.63714	-2.0772	0	0.000706
PPI_BIOGRID_M734	30	11	-0.69304	-2.0258	0	0.00113
PPI_BIOGRID_M848	22	11	-0.67958	-1.9924	0	0.001177
PPI_BIOGRID_M857	14	13	-0.83146	-2.0371	0	0.001256
PPI_BIOGRID_M581	56	23	-0.63221	-2.0062	0	0.001284
PPI_BIOGRID_M172	31	14	-0.63806	-1.9488	0	0.001507
PPI_BIOGRID_M544	20	12	-0.7468	-1.9646	0	0.001521
PPI_BIOGRID_M438	16	6	-0.74768	-1.9459	0	0.001589
PPI_BIOGRID_M597	13	6	-0.85103	-1.9511	0	0.001614
PPI_BIOGRID_M309	238	89	0.83885	1.6286	0	0.003523
PPI_BIOGRID_M185	32	21	-0.63805	-1.8991	0	0.003822
PPI_BIOGRID_M702	15	8	-0.76267	-1.8672	0	0.006592
PPI_BIOGRID_M722	46	24	-0.58535	-1.8575	0	0.007286
PPI_BIOGRID_M189	11	7	-0.86049	-1.8475	0	0.008616
PPI_BIOGRID_M717	23	12	-0.67161	-1.8398	0	0.008732
PPI_BIOGRID_M583	69	27	-0.54002	-1.8412	0	0.008744
PPI_BIOGRID_M951	10	5	-0.81538	-1.8293	0.008929	0.010809
PPI_BIOGRID_M190	11	7	-0.79575	-1.8176	0.016949	0.012359
PPI_BIOGRID_M819	10	8	-0.80619	-1.8117	0	0.012656

From the results of KEGG pathway GSEA (Table 1), Primary immunodeficiency, Th1 and Th2 cell differentiation, Chemokine signaling pathway, T cell receptor signaling pathway, Th17 cell differentiation, and Fc gamma R-mediated phagocytosis were associated with immunity. From the results of Panther Pathway GSEA (Table 2), T cell activation, B cell activation, Inflammation mediated by chemokine and cytokine signaling pathway, Interleukin signaling pathway, and Toll receptor signaling pathway were associated with immunity. From the results of Reactome Pathway GSEA (Table 3), Defensins, Translocation of ZAP-70 to Immunological synapse, Generation of second messenger molecules, Costimulation by the CD28 family, PD-1 signaling, Interleukin-2 family signaling, Interleukin-10 signaling, Interleukin-3, Interleukin-5 and GM-CSF signaling, DAP12 interactions, Immunoregulatory interactions between a Lymphoid and a non-Lymphoid cell, Phosphorylation of CD3 and TCR zeta chains, DAP12 signaling, Interleukin receptor SHC signaling, Antigen activates B Cell Receptor (BCR) leading to generation of second messengers, RHO GTPases Activate NADPH Oxidases, Chemokine receptors bind chemokines, Interferon-gamma signaling, and Regulation of actin dynamics for phagocytic cup formation were associated with immunity. From the results of Wikipathway GSEA analysis (Table 4), T-Cell antigen Receptor (TCR) Signaling Pathway, T-Cell antigen Receptor (TCR) pathway during *Staphylococcus aureus* infection, Allograft Rejection, IL-3 Signaling Pathway, Type II interferon signaling (IFNG), Interactions between immune cells and microRNAs in the tumor microenvironment, Cancer immunotherapy by PD-1 blockade, IL-2 Signaling Pathway, IL-9 Signaling Pathway, IL-7 Signaling Pathway, Macrophage markers, Chemokine signaling pathway, Selective expression of chemokine receptors during T-cell polarization, Cancer immunotherapy by CTLA4 blockade, T-Cell Receptor and Co-stimulatory Signaling, B Cell Receptor Signaling Pathway, Inflammatory Response Pathway, and IL-5 Signaling Pathway were associated with immunity. From the results of Gene ontology Biological Process GSEA (Table 5), the GO terms cellular defense response, interleukin-2 production, interferon-gamma production, leukocyte proliferation, lymphocyte activation involved in immune response, leukocyte cell-cell adhesion, myeloid dendritic cell activation, adaptive immune response, T cell activation, interleukin-4 production, cytokine metabolic process, tumor necrosis factor superfamily cytokine production, response to chemokine, natural killer cell activation, regulation of leukocyte activation, B cell activation, immune response-regulating signaling pathway, and interleukin-10 production were associated with immunity. From the results of the Gene ontology Cellular Component GSEA (Table 6), the GO terms MHC protein complex, immunological synapse, and mast cell granule were associated with immunity. From the results of Gene ontology Molecular Function GSEA (Table 7–10) the GO terms MHC protein binding, cytokine receptor activity, immunoglobulin binding, antigen binding, and cytokine binding were associated with immunity.

### The relationship between TILs, immunostimulators, MHC molecules, chemokines, and chemokine receptors and the expression and DNA methylation of *ARHGAP30* in lung adenocarcinoma

### The relationship between ARHGAP30 expression and TILs, immunostimulators, MHC molecules, chemokines, and chemokine receptors in LUAD

**Figure 8:**
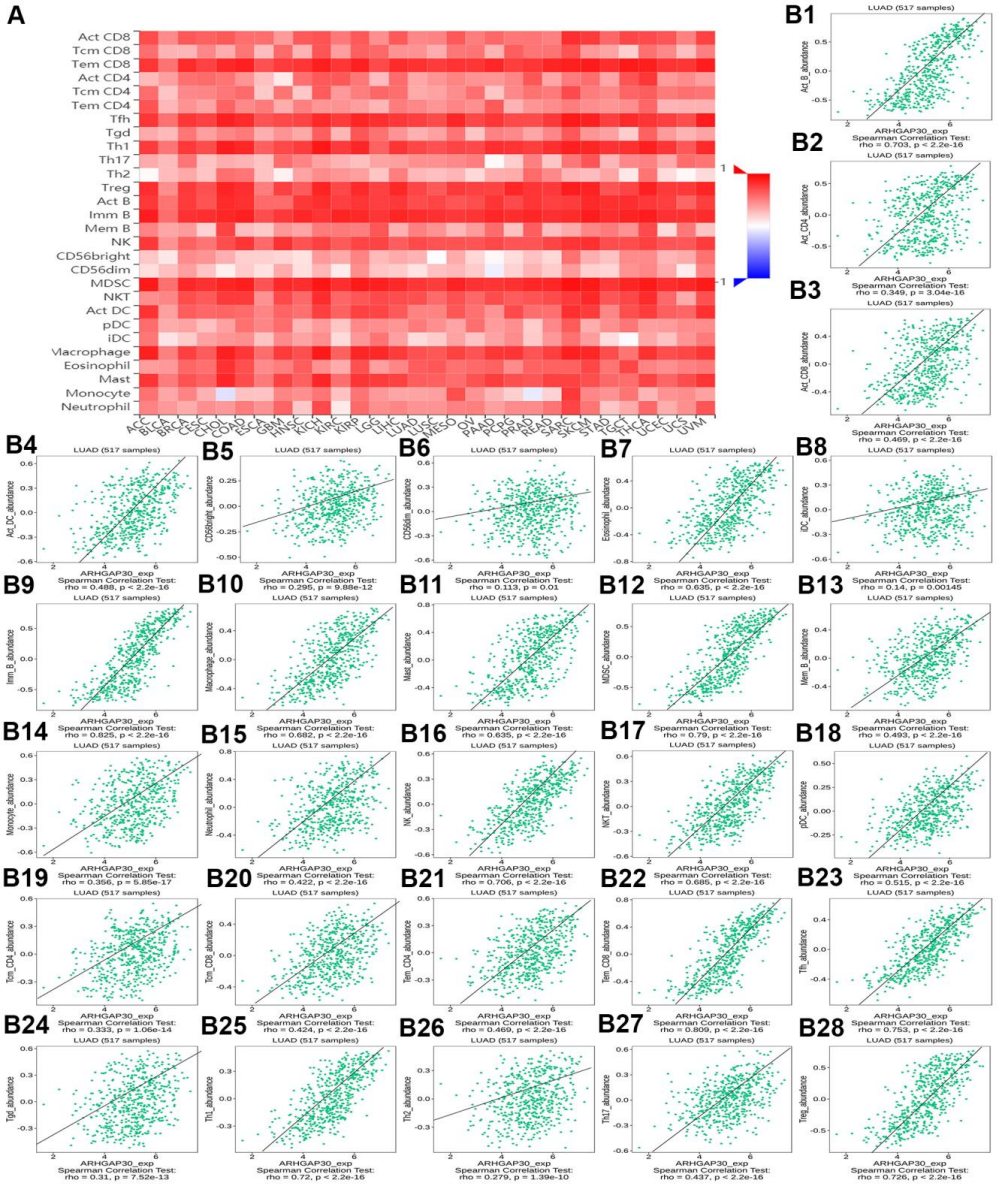
**The correlation between the abundance of tumor-infiltrating lymphocytes (TILs) and expression of *ARHGAP30*.** (**A**) Heat map of the relationship between the abundance of TILs and *ARHGAP30* expression. (**B1**–**B28**) Scatter plots showing the positive correlation between *ARHGAP30* expression and TILs in the treatment of lung adenocarcinoma. Act_CD8, Activated CD8 T cell; Tcm_CD8, Central memory CD8 T cell; Tem_CD8, Effector memory CD8 T cell; Act_CD4, Activated CD4 T cell; Tcm_CD4, Central memory CD4 T cell; Tem_CD4, Effector memory CD4 T cell; Tgd, Gamma delta T cell; Tfh, T follicular helper cell; Th1, Type 1 T helper cell; Th17, Type 17 T helper cell; Th2, Type 2 T helper cell; Treg, Regulatory T cell; MDSC, Myeloid derived suppressor cell; Act_B, Activated B cell; Imm_B, Immature B cell; Mem_B, Memory B cell; NK, Natural killer cell; CD56brigh, CD56bright natural killer cell; CD56dim, CD56dim natural killer cell; NKT, Natural killer T cell; Act_DC, Activated dendritic cell; iDC, Immature dendritic cell; pDC, Plasmacytoid dendritic cell; Mast, Mast cell.

**Figure 9:**
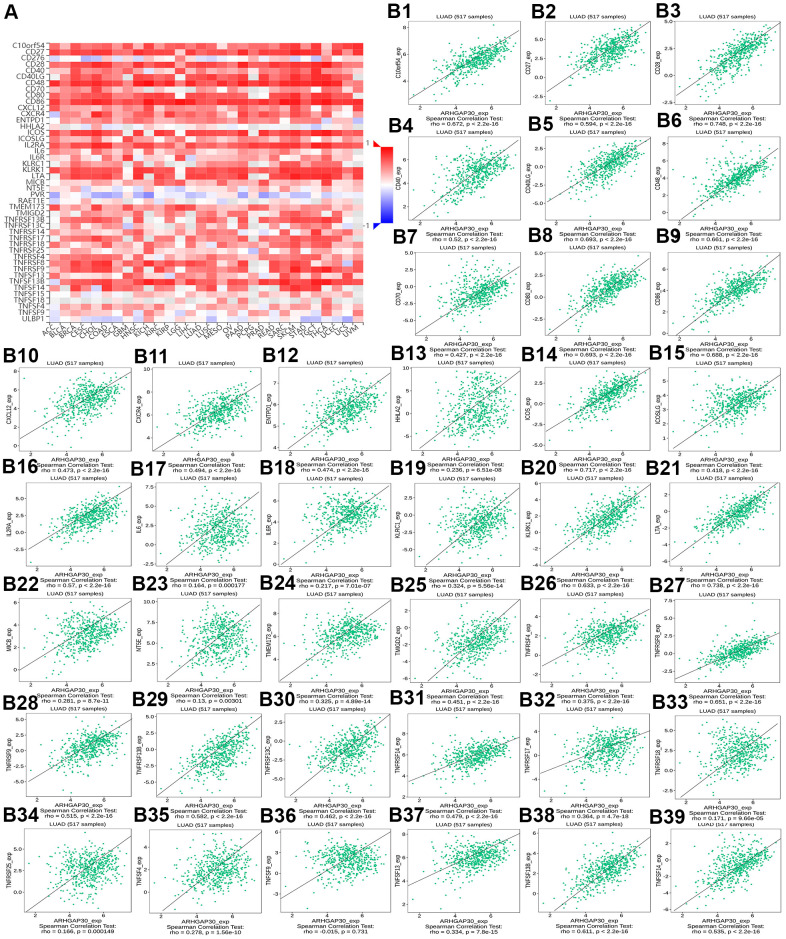
**The correlation between the abundance of tumor-infiltrating lymphocytes (TILs) and the methylation of *ARHGAP30*.** (**A**) Heat map of the relationship between the abundance of TILs abundance and *ARHGAP30* DNA methylation. (**B1**–**B39**) Scatter plots showing the negative correlation between *ARHGAP30* DNA methylation and TILs in the treatment of lung adenocarcinoma. Act_CD8, Activated CD8 T cell; Tcm_CD8, Central memory CD8 T cell; Tem_CD8, Effector memory CD8 T cell; Act_CD4, Activated CD4 T cell; Tcm_CD4, Central memory CD4 T cell; Tem_CD4, Effector memory CD4 T cell; Tgd, Gamma delta T cell; Tfh, T follicular helper cell; Th1, Type 1 T helper cell; Th17, Type 17 T helper cell; Th2, Type 2 T helper cell; Treg, Regulatory T cell; MDSC, Myeloid derived suppressor cell; Act_B, Activated B cell; Imm_B, Immature B cell; Mem_B, Memory B cell; NK, Natural killer cell; CD56brigh, CD56bright natural killer cell; CD56dim, CD56dim natural killer cell; NKT, Natural killer T cell; Act_DC, Activated dendritic cell; iDC, Immature dendritic cell; pDC, Plasmacytoid dendritic cell; Mast, Mast cell.

**Figure 10:**
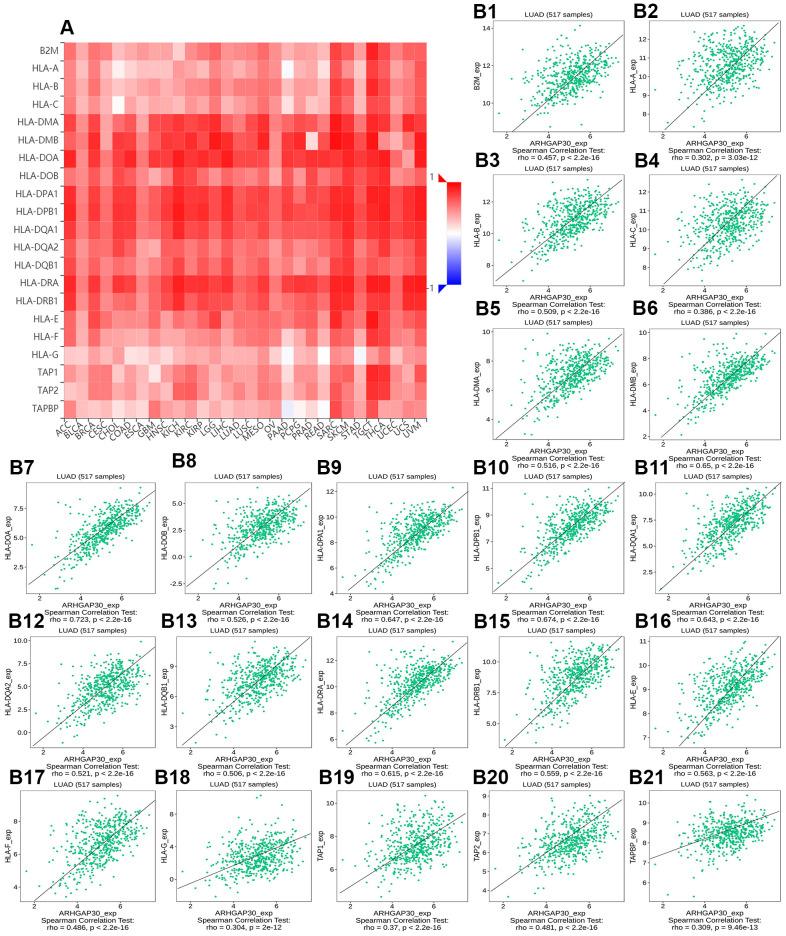
**The correlation between the expression of *ARHGAP30* and immune inhibitors.** (**A**) Heat map of Spearman correlations between *ARHGAP30* expression and immune inhibitors across human cancers. (**B1**–**B21**) Scatter plots showing the positive correlation between *ARHGAP30* expression and immune inhibitors in the treatment of lung adenocarcinoma.

**Figure 11:**
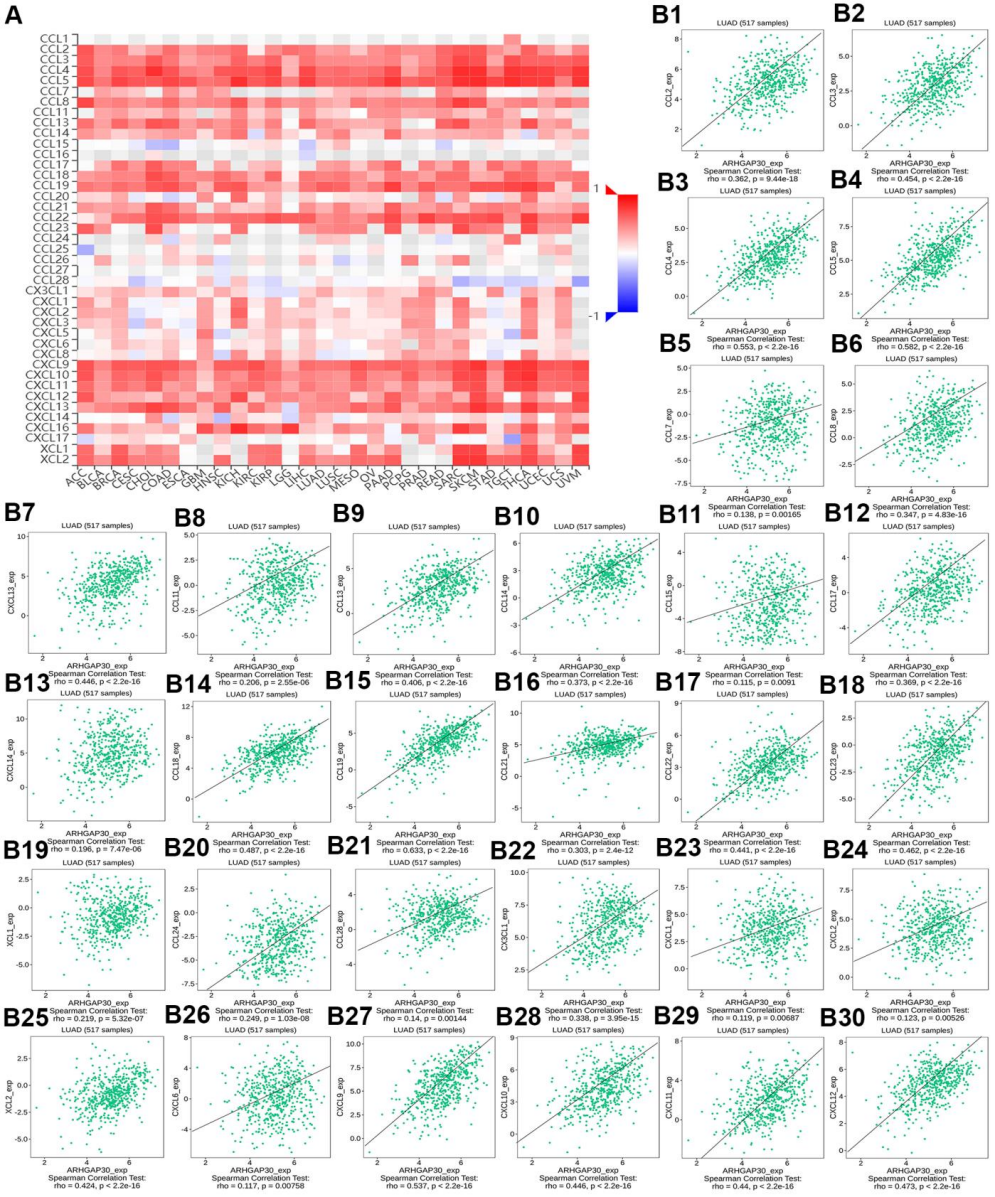
**The correlation between the DNA methylation of *ARHGAP30* and immune inhibitors.** (**A**) Heat map of Spearman correlations between DNA methylation of *ARHGAP30* and immune inhibitors across human cancers. (**B1**–**B30**) Scatter plots showing the negative correlation between DNA methylation of *ARHGAP30* and immune inhibitors in the treatment of lung adenocarcinoma.

**Figure 12:**
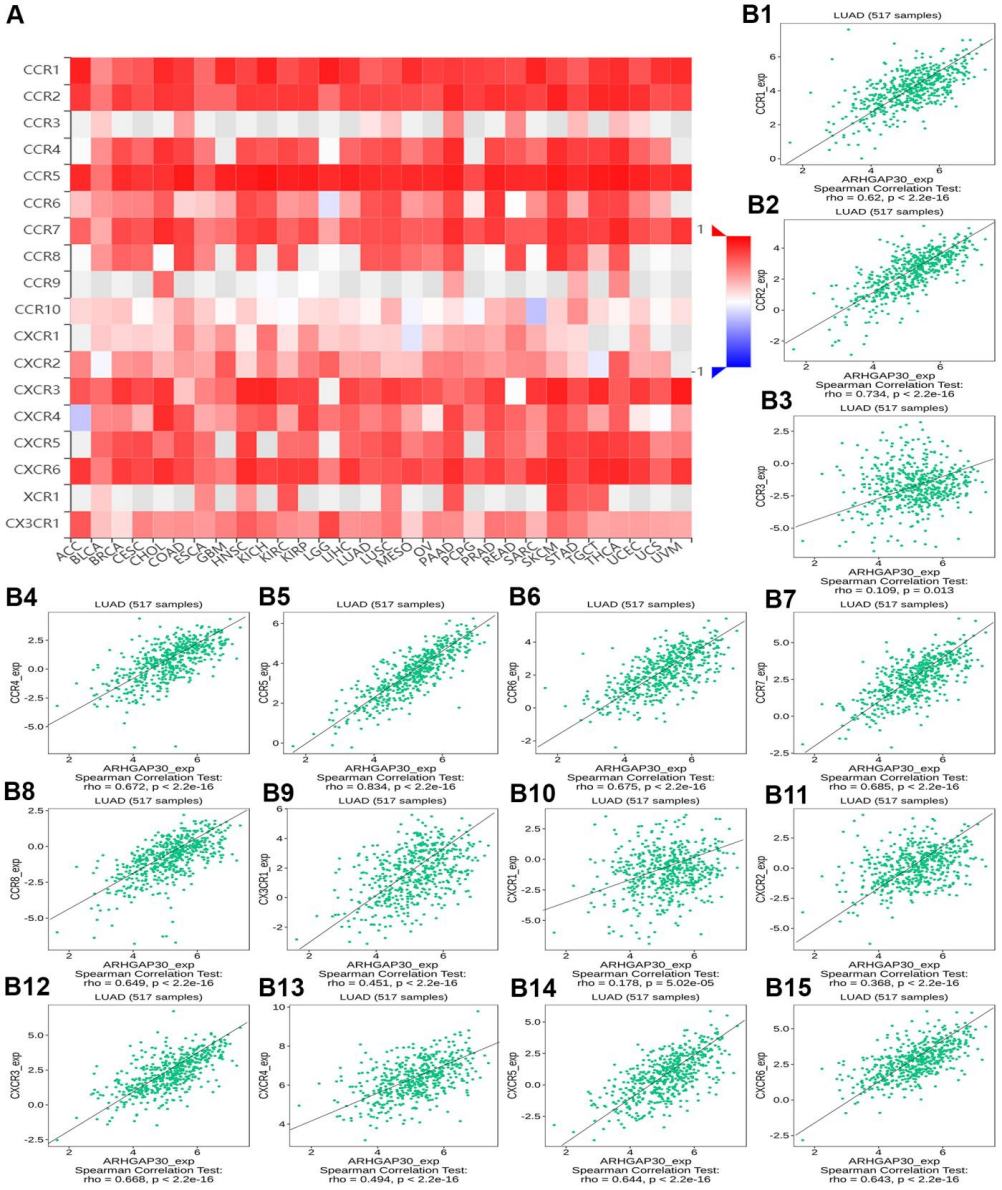
**The correlation between the expression of *ARHGAP30* and immunostimulators.** (**A**) Heat map of Spearman correlations between *ARHGAP30* expression and immunostimulators across human cancers. (**B1**–**B15**) Scatter plots showing the positive correlation between *ARHGAP30* expression and immunostimulators in the treatment of lung adenocarcinoma.

Figures 8A, 9A, 10A, 11A, 12A, respectively, show heat maps of the relationship between the abundance of TILs, immunostimulators, MHC molecules, chemokines, and chemokine receptors and the expression of *ARHGAP30*. These heatmaps were mostly red, indicating that most of the TILs, immunostimulators, MHC molecules, chemokines, and chemokine receptors correlated positively with the expression of *ARHGAP30*. Also, dark red areas indicated that some of them had a strong positive correlation with the expression of *ARHGAP30*.

Figure 8B1–8B28 show scatter plots of the relations the abundance of TILs and *ARHGAP30* expression. The results showed that effector memory CD8 T cells, T follicular helper cells, type 1 T helper cells, regulatory T cells, myeloid derived suppressor cells, activated B cells, immature B cells, natural killer cells, natural killer T cells, macrophages, eosinophils, and mast cells showed a strong positive correlation with the expression of *ARHGAP30* in LUAD (Spearman correlation coefficient, r > 0.6; p value < 0.01). Figure 9B1–9B39 shows scatter plots of the relationship between the abundance of immunostimulators and *ARHGAP30* expression. The results showed that C10orf54, CD28, CD40LG, CD48, CD80, CD86, ICOS, KLRK1, LTA, and TNFRSF8 showed a strong positive correlation with the expression of *ARHGAP30* in LUAD (Spearman correlation coefficient, r > 0.6; p value < 0.01). Figure 10B1–10B21 show scatter plots of the relationship between the abundance of MHC molecules and *ARHGAP30* expression. The results showed that HLA-DMB, HLA-DOA, HLA-DPA1, HLA-DPB1, HLA-DQA1, and HLA-DRA showed a strong positive correlation with the expression of *ARHGAP30* in LUAD (Spearman correlation coefficient, r > 0.6; p value < 0.01). Figure 11B1–11B30 show scatter plots of the relationship between the abundance of chemokines and *ARHGAP30* expression. The results showed that CCL19 showed a strong positive correlation with the expression of *ARHGAP30* in LUAD (Spearman correlation coefficient, r > 0.6; p value < 0.01). Figure 12B1–12B15 show scatter plots of the relationship between the abundance of chemokine receptors and *ARHGAP30* expression. The results showed that CCR1, CCR2, CCR4, CCR5, CCR6, CCR7, CCR8, CXCR3, CXCR5, and CXCR6 showed a strong positive correlation with the expression of *ARHGAP30* in LUAD (Spearman correlation coefficient, r > 0.6; p value < 0.01).

### The relationship between DNA methylation of ARHGAP30 and TILs, immunostimulators, MHC molecules, chemokines, and chemokine receptors in LUAD

**Figure 13:**
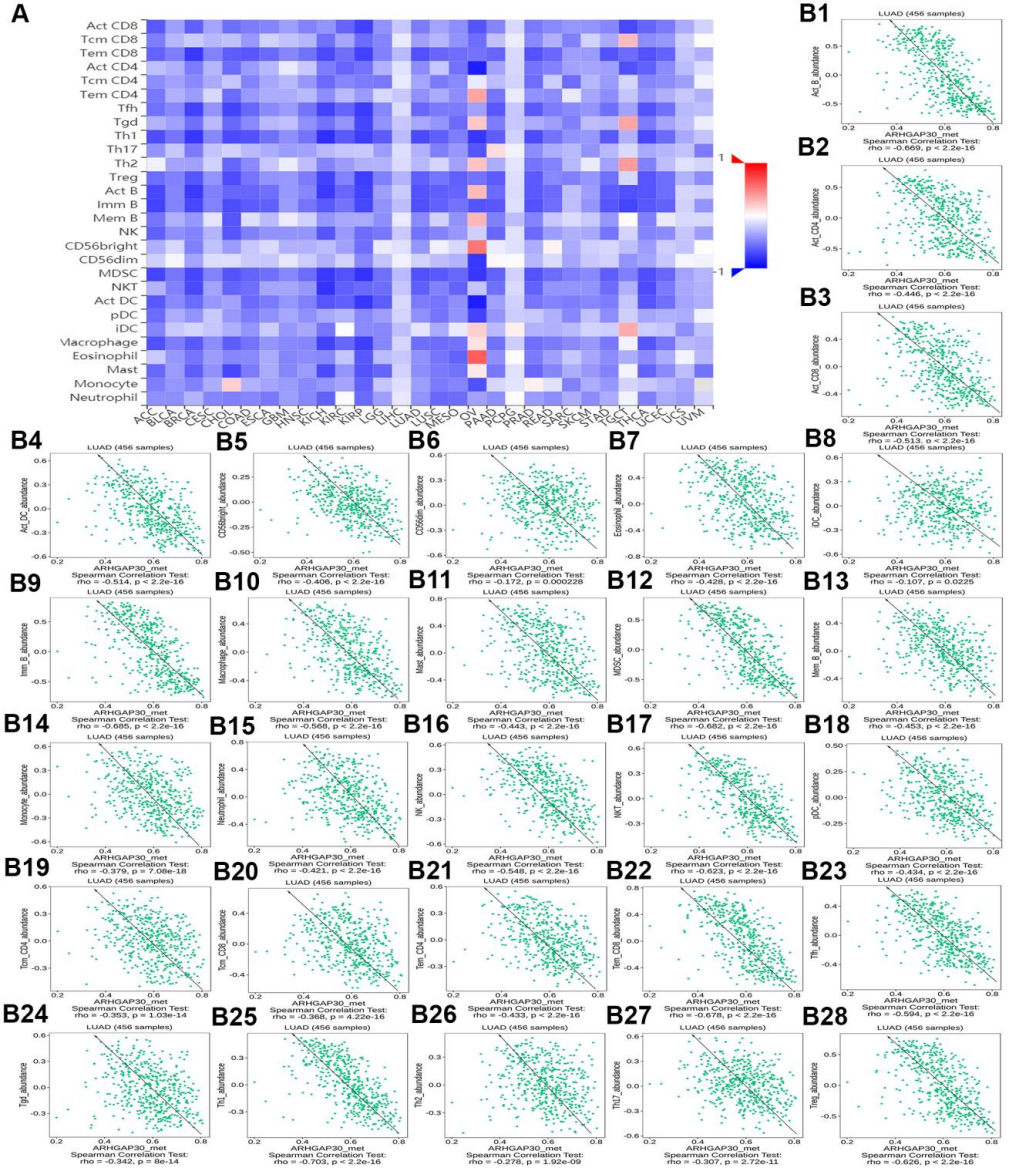
**The correlation between the DNA methylation of *ARHGAP30* and Immunostimulators.** (**A**) Heat map of Spearman correlations between DNA methylation of *ARHGAP30* and immunostimulators across human cancers. (**B1**–**B28**) Scatter plots showing the negative correlation between DNA methylation of *ARHGAP30* and immunostimulators in the treatment of lung adenocarcinoma.

Figures 13A and Supplementary Figures 10A, 11A, 12A, 13A, respectively, show heat maps of the relationship between TILs, immunostimulators, MHC molecules, chemokines, and chemokine receptors and DNA methylation of *ARHGAP30*. The results showed that in LUAD, most of them were blue, indicating that most of the TILs, immunostimulators, MHC molecules, chemokines, and chemokine receptors correlated negatively with DNA methylation of *ARHGAP30*. Also, some of them were very dark blue, indicating that they had a strong negative correlation with DNA methylation of *ARHGAP30*.

Figure 13B1–13B28 show scatter plots of the relationship between the abundance of TILs and DNA methylation of *ARHGAP30*. The results showed that activated B cell, immature B cell, myeloid derived suppressor cell, natural killer T cell, effector memory CD8 T cell, type 1 T helper cell, and regulatory T cell had a strong negative correlation with the DNA methylation of *ARHGAP30* in LUAD (Spearman correlation coefficient, r < - 0.6; p value < 0.01). Supplementary Figure 10B1–10B39 show scatter plots of the relationship between the abundance of immunostimulators and DNA methylation of *ARHGAP30*. The results showed that CD28, CD48, LTA, and TNFRSF8 had a strong negative correlation with the DNA methylation of *AGHGAP30* in LUAD (Spearman correlation coefficient, r < - 0.6; p value < 0.01). Supplementary Figure 11B1–11B21 show scatter plots of the relationship between the abundance of MHC molecules and DNA methylation of *ARHGAP30*. Supplementary Figure 12B1–12B30 show scatter plots of the relationship between the abundance of chemokines and DNA methylation of *ARHGAP30*. Supplementary Figure 13B1–13B15 show scatter plots of the relationship between the abundance of chemokine receptors and DNA methylation of *ARHGAP30*. The results showed that CCR5 and CCR6 had a strong negative correlation with the DNA methylation of *ARHGAP30* in LUAD (Spearman correlation coefficient, r < - 0.6; p value < 0.01).

## DISCUSSION AND CONCLUSIONS

In this study, we showed that the expression of *ARHGAP30* in LUAD tissues was significantly lower than that in normal tissues. There were differences in *ARHGAP30* mRNA expression levels in patients with LUAD with different sexes, cancer stages, and nodal metastatic status (Figure 1). The expression of *ARHGAP30* in LUAD tissues was significantly lower in the presence of *KEAP1* and *STK11* mutations. The correlation between DNA methylation of *ARHGAP30* and its mRNA expression levels was considerably higher in LUAD tissues than in normal tissues (Figure 2). There are some studies on the differential expression of *ARHGAP30* in cancer [8, 34, 35]. The high DNA methylation level of *ARHGAP30* might also be one of the reasons for the decreased *ARHGAP30* expression in LUAD tissues. Genetic mutations in *KEAP1* and *STK11* might also be another reason for decreased expression of *ARHGAP30* in LUAD tissues. These were not reported in previous studies.

Patients with LUAD with low *ARHGAP30* expression had a significantly better prognosis than those with high *ARHGAP30* expression (Figure 3). A study by Mao and Tong [35] also supports this point. Although some prognostic molecular markers have been found in patients with LUAD [36–43], *ARHGAP30* might be developed as a molecular marker to evaluate the prognosis of patients with LUAD after surgery or in patients with advanced disease. We identified genes, miRNAs, and lncRNAs that were highly associated with *ARHGAP30* in LUAD (Figures 4–6), which could provide new ideas and targets for epigenetic studies of *ARHGAP30* in LUAD.

**Figure 14:**
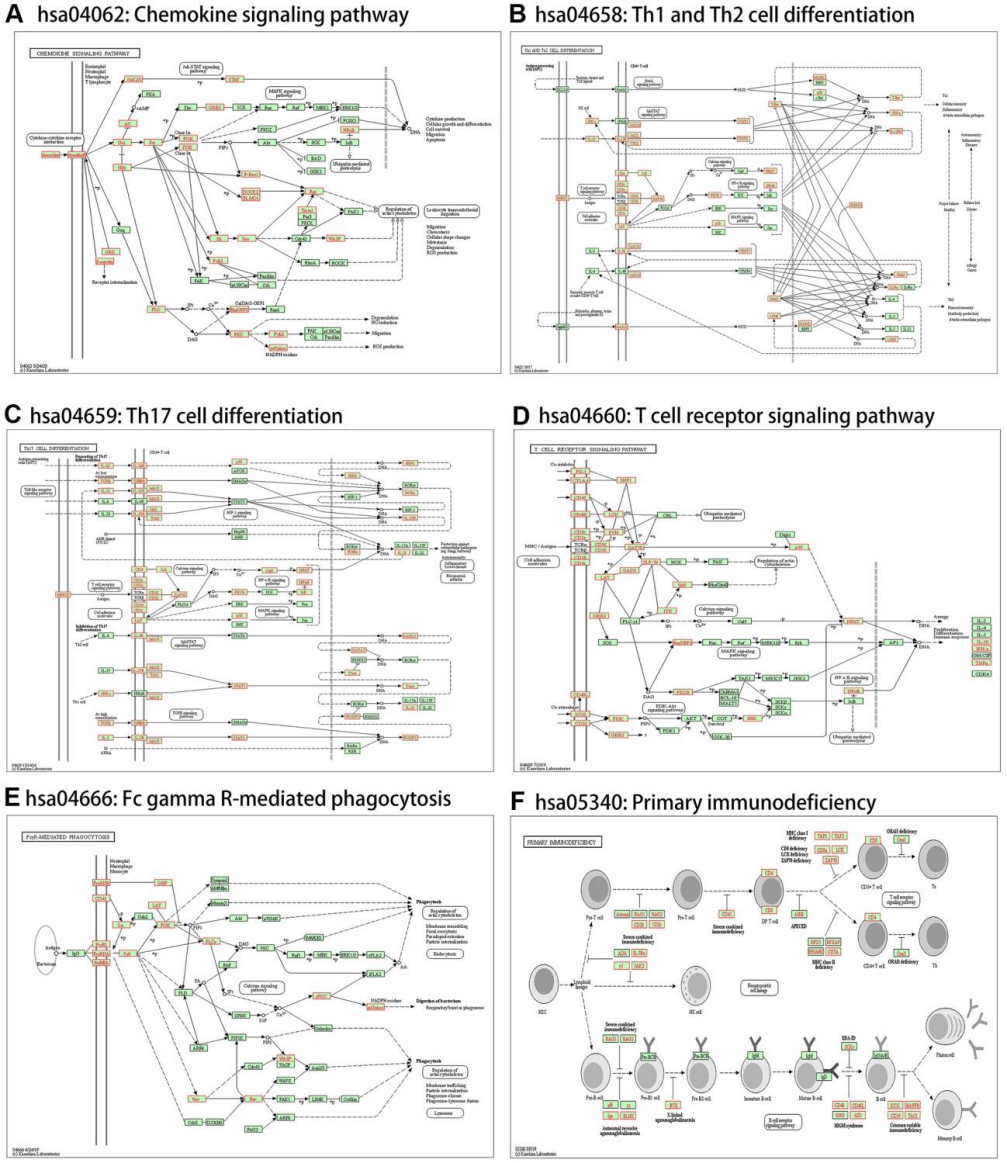
**Immune-related statistically significant KEGG pathway annotations.** (**A**) Chemokine signaling pathway (hsa04062). (**B**) Th1 and Th2 cell differentiation (hsa04658). (**C**) Th17 cell differentiation (hsa04659). (**D**) T cell receptor signaling pathway (hsa04660). (**E**) Fc gamma R-mediated phagocytosis (hsa04666). (**F**) Primary immunodeficiency (hsa05340). Red denotes leading-edge genes; green denotes the remaining genes.

We identified many pathways related to tumor immunity from the enrichment results of KEGG Pathway, Panther Pathway, Reactome Pathway, and Wikipathway (Figures 7, 14 and Supplementary Figures 1–3). Recent studies have demonstrated a close relationship between Rho GTPases and the development and metastasis of a variety of human tumors [7]. KEGG pathways included Primary immunodeficiency, Th1 and Th2 cell differentiation, Chemokine signaling pathway, T cell receptor signaling pathway, Th17 cell differentiation, and Fc gamma R-mediated phagocytosis. Panther pathways included T cell activation, B cell activation, Inflammation mediated by chemokine and cytokine signaling pathway, Interleukin signaling pathway and Toll receptor signaling pathway. Reactome Pathways Defensins, Translocation of ZAP-70 to Immunological synapse, Generation of second messenger molecules, Costimulation by the CD28 family, PD-1 signaling, Interleukin-2 family signaling, Interleukin-10 signaling, Interleukin-3, Interleukin-5 and GM-CSF signaling, DAP12 inter-actions, Immunoregulatory interactions between a Lymphoid and a non-Lymphoid cell, Phosphorylation of CD3 and TCR zeta chains, DAP12 signaling, Interleukin receptor SHC signaling, Antigen activates B Cell Receptor (BCR) leading to generation of second messengers, RHO GTPases Activate NADPH Oxidases, Chemokine receptors bind chemokines, Interferon gamma signaling and Regulation of actin dynamics for phagocytic cup formation. Wikipathways included T-Cell antigen Receptor (TCR) Signaling Pathway, T-Cell antigen Receptor (TCR) pathway during Staphylococcus aureus infection, Allograft Rejection, IL-3 Signaling Pathway, Type II interferon signaling (IFNG), Interactions between immune cells and microRNAs in tumor microenvironment, Cancer immunotherapy by PD-1 blockade, IL-2 Signaling Pathway, IL-9 Signaling Pathway, IL-7 Signaling Pathway, Macrophage markers, Chemokine signaling pathway, Selective expression of chemokine receptors during T-cell polarization, Cancer immunotherapy by CTLA4 blockade, T-Cell Receptor and Co-stimulatory Signaling, B Cell Receptor Signaling Pathway, Inflammatory Response Pathway, and IL-5 Signaling Pathway.

We further observed that the levels of TILs, immunostimulators, MHC molecules, chemokines, chemokine receptors and *ARHGAP30* expression correlated positively in LUAD (Figures 8–13); however, these factors correlated negatively with the DNA methylation level of *ARHGAP30* (Supplementary Figures 10–13). Anti-tumor immunotherapy is promising treatment modality in the fight against tumors; however, previous application found that its efficacy was not as good as expected. Through in-depth studies, it has been found that immune tolerance in the tumor microenvironment might be the most important reason leading to the unsatisfactory effects of immunotherapy [44, 45]. Defects in the development or function of CD8^+^ cytotoxic T lymphocytes (CTLs), CD4^+^ Th1 helper T cells, or natural killer (NK) cells all lead to more frequent tumorigenesis and/or more rapid growth [46]. Immunostimulators could accumulate in tumors and significantly inhibit tumor growth [47]. A tumor can escape T cell reactions by losing major histocompatibility complex (MHC) molecules [48]. Chemokines and chemokine receptors mediate the host response to cancer by directing leukocytes into the tumor microenvironment [49, 50]. Our results supported the above points. *ARHGAP30* expression correlated positively with TILs, immunostimulators, MHC molecules, chemokines, and chemokine receptors in LUAD (Figures 8–12), which might be related to the significantly reduced *ARHGAP30* expression in LUAD. Levels of TILs, immunostimulators, MHC molecules, chemokines, and chemokine receptors were decreased in LUAD. Reduced or functional defects in tumor immune function result in more frequent occurrence and more rapid proliferation and growth of LUAD.

Therefore, we proposed that DNA methylation of *ARHGAP30* and mutations in *KEAP1* and *STK11* genes inhibit *ARHGAP30* expression in LUAD. Decreased *ARHGAP30* expression might inhibit TILs, immunostimulators, MHC molecules, chemokines, and chemokine receptors in lung adenocarcinoma through pathways identified in the enrichment analysis, which in turn inhibits tumor immunity and ultimately promotes the formation and growth of LUAD.

Our study is the first to perform prognostic analysis and evaluation of *ARHGAP30* in patients with LUAD, to carry out GSEA of *ARHGAP30*, and to investigate the relationship between *ARHGAP30* and TILs, immunostimulators, MHC molecules, chemokines, and chemokine receptors in LUAD. These findings have important implications for the diagnosis, prognostic evaluation, and cancer immunotherapy of patients with LUAD Our study was limited by a lack of further experimental validation. We could also assess the relationship of *ARHGAP30* with other types of lung cancer to determine the specific role of *ARHGAP30* expression in the diagnosis and treatment of different types of lung cancer.

Overall, our results suggest that DNA methylation of *ARHGAP30*, as well as mutations in *KEAP1* and *STK11*, inhibit *ARHGAP30* expression in LUAD, which in turn promotes LUAD formation and growth through multiple pathways that suppress tumor infiltrating immunity, thus contributing to poor prognosis of patients with LUAD.

## MATERIALS AND METHODS

We used the Oncomine 4.5 [10] database to analyze the differential expression of *ARHGAP30* in various cancers and in the Hou lung, Selamat lung, and Okayama lung adenocarcinoma datasets. We used the SurvExpress [11] database to analyze the differential expression of *ARHGAP30* in two lung adenocarcinoma datasets. We used the GEPIA [12] database to analyze the differential expression of *ARHGAP30* in lung adenocarcinoma. We used the Warner [13] database to explore the abundance of different exons of the *ARHGAP30* gene in normal and tumor tissues of patients with LUAD. We used the Ualcan [14] database to analyze the differences of *ARHGAP30* mRNA expression in subgroups of patients with lung adenocarcinoma patients according to sample type, individual cancer stage, ethnicity, sex, age, smoking habit, nodal metastasis status, and *TP53* mutation status. We used the Ualcan [14] and CPTAC [15] databases to analyze the differential expression of *ARHGAP30* protein in patients with LUAD stratified by sample type, individual cancer stage, ethnicity, sex, age, weight, tumor grade, and tumor histology.

We used the TCGA portal [16] database to analyze the differential expression of *ARHGAP30* after highly mutated gene mutation. We also used the TCGA portal database to analyze the correlation between *ARHGAP30* gene expression and DNA methylation in primary tumors and normal tissue samples. We analyzed the mRNA expression of *ARHGAP30* in LUAD before and after mutation of highly mutated genes (*KEAP1*, *STK11*) using the Linkedomics [17] database. We analyzed the heatmap of *ARHGAP30* methylation in lung adenocarcinoma using the MethSurv [18] database. The Kaplan–Meier plots of patients with LUAD assessed using different *ARHGAP30* methylation probes (cg07837534 and cg00045607) were analyzed.

We used GEPIA [12], Oncolnc [19], Ualcan [14], UCSC [20], TCGAportal [16], TISIDB [21], KMplot [22], TIMER [23], Linkedomics [17], and PrognoScan [24] databases to analyze the overall survival (OS) curves for patients with LUAD. We used the GEPIA [12] database to analyze the disease-free survival (DFS) curves for patients with LUAD (in months and days, respectively). We used the PrognoScan database to analyze the recurrence-free survival (RFS) curves in patients with LUAD.

We analyzed the genes and mRNAs that were highly associated with *ARHGAP30* in LUAD using the Linkedomics [17] database and obtained the corresponding volcano plots, heat plots, and scatter plots. We analyzed the lncRNAs that were highly associated with *ARHGAP30* in LUAD using the TANRIC [25] database and obtained the corresponding scatter plots and survival curves.

We used the TISIDB [21] database to analyze the relationship between TILs, immunostimulators, MHC molecules, chemokines, chemokine receptors and the expression and DNA methylation of *ARHGAP30* in LUAD.

### Statistical methods

We used a t-test to analyze the differential expression levels of *ARHGAP30* in normal and tumor samples. We analyzed the DNA methylation expression levels of *ARHGAP30* in normal and tumor samples using the Wilcoxon rank sum test. We used Pearson correlation [51–54] to analyze *ARHGAP30*-associated genes, miRNAs, and lncRNAs. We performed survival analysis and plotted Kaplan–Meier curves for *ARHGAP30*. We performed gene set enrichment analysis (GSEA) [26] of *ARHGAP30* for KEGG Pathway [27], Panther Pathway [28], Reactome Pathway [29], Wikipathway [30], Gene ontology Biological Process [31, 32], Gene ontology Cellular Component [31, 32], Gene ontology Molecular Function [31, 32], Kinase Target Network, Transcription Factor Network, and PPI BIOGRID Network [33].

### Ethics approval and declaration

This study was approved by the academic ethics review board of the Second Affiliated Hospital of Nanchang University. Human participants and research animals were not involved in this study. All software applications are freely and publicly available without custom code. All data in this article were obtained from publicly available databases, and all the data and pictures in this article are authorized.

## Supplementary Material

Supplementary Figures

Supplementary Tables
