# Supplementary material for: DNA methylation of ARHGAP30 is negatively associated with ARHGAP30 expression in lung adenocarcinoma, which reduces tumor immunity and is detrimental to patient survival
Source: Aging (Albany NY). 2021 Dec 15;13(24):25799–845. doi: 10.18632/aging.203762 (PMC8751594; doi:10.18632/aging.203762)
Supplement: Supplementary Figures [file aging-13-203762-s001.pdf]

## SUPPLEMENTARY FIGURES

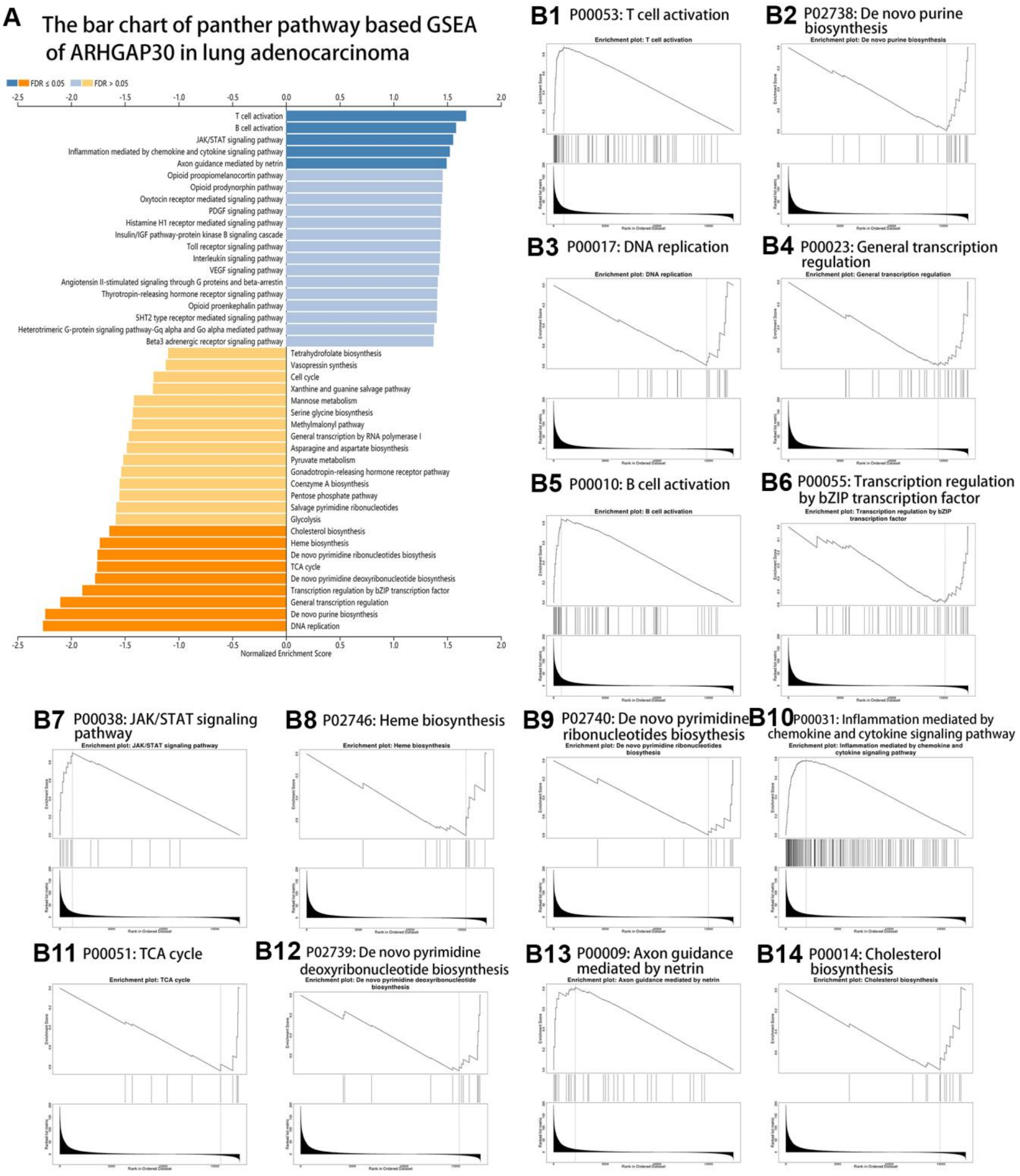


**Supplementary Figure 1. Panther pathway-based GSEA of *ARHGAP30* in lung adenocarcinoma (LUAD).** (**A**) Bar chart of Panther Pathway-based GSEA of *ARHGAP30* in LUAD (FDR < 0.05). (**B1**–**B14**) GSEA enrichment analysis Plots of 14 tumor immune-related Panther Pathway gene sets (FDR < 0.05).

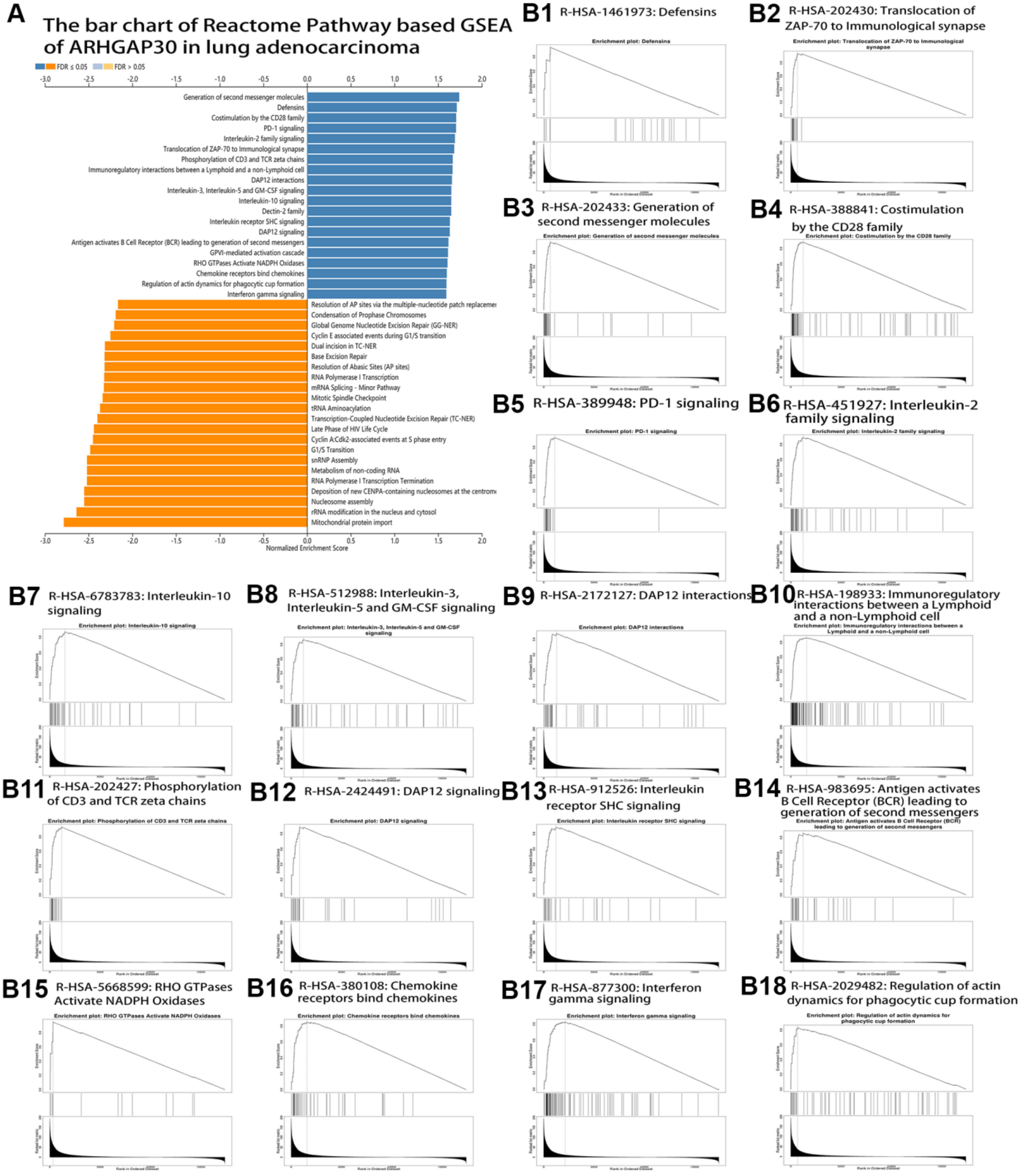


**Supplementary Figure 2. Reactome pathway-based GSEA of *ARHGAP30* in lung adenocarcinoma (LUAD).** (**A**) Bar chart of Reactome Pathway-based GSEA of *ARHGAP30* in LUAD (FDR < 0.05). (**B1**–**B18**) GSEA enrichment analysis Plots of 18 tumor immune-related Reactome Pathway gene sets (FDR < 0.05).

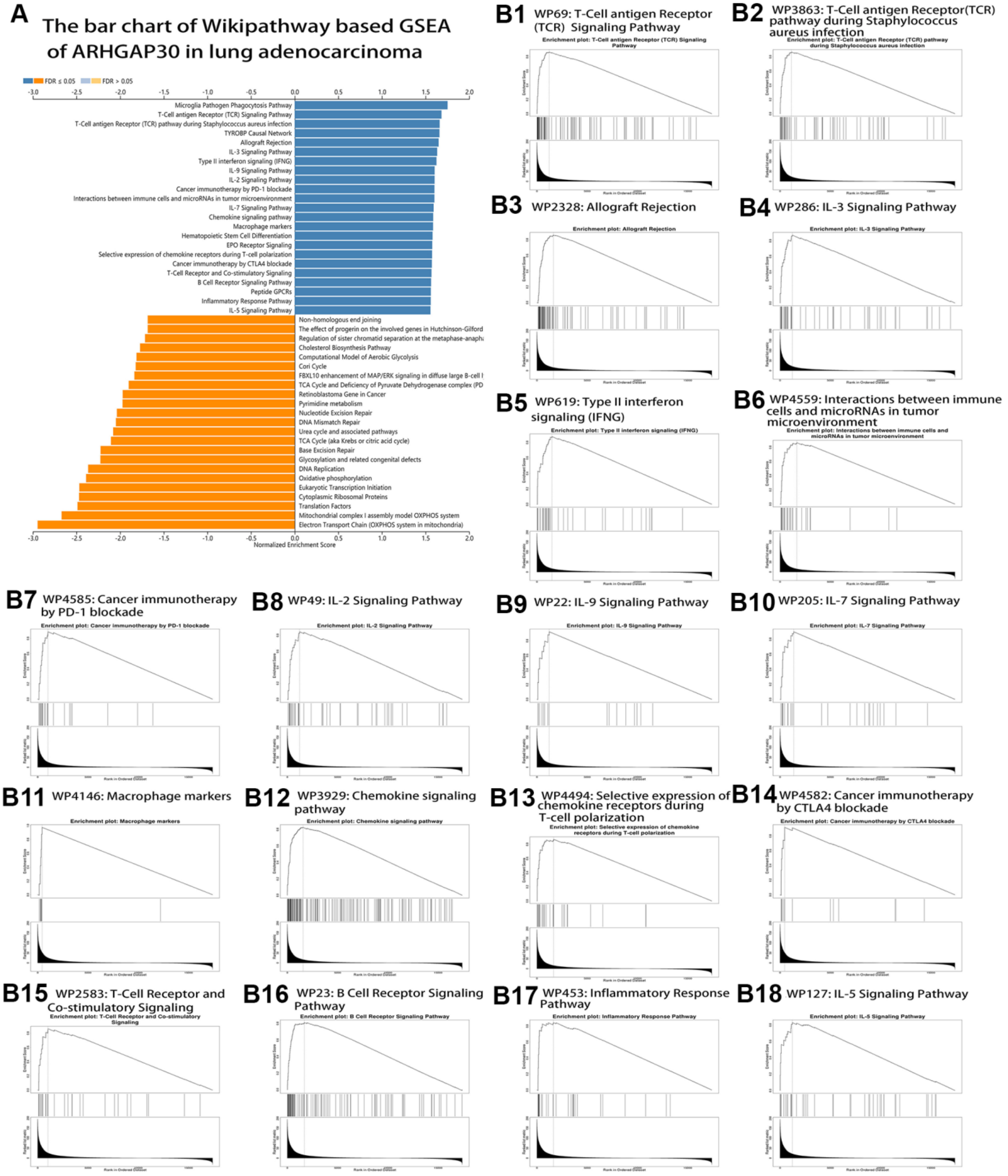


**Supplementary Figure 3. Wikipathway-based GSEA of *ARHGAP30* in lung adenocarcinoma (LUAD).** (**A**) Bar chart of Wikipathway-based GSEA of *ARHGAP30* in LUAD (FDR < 0.05). (**B1**–**B18**) GSEA enrichment analysis Plots of 18 tumor immune-related Wikipathway gene sets (FDR < 0.05).

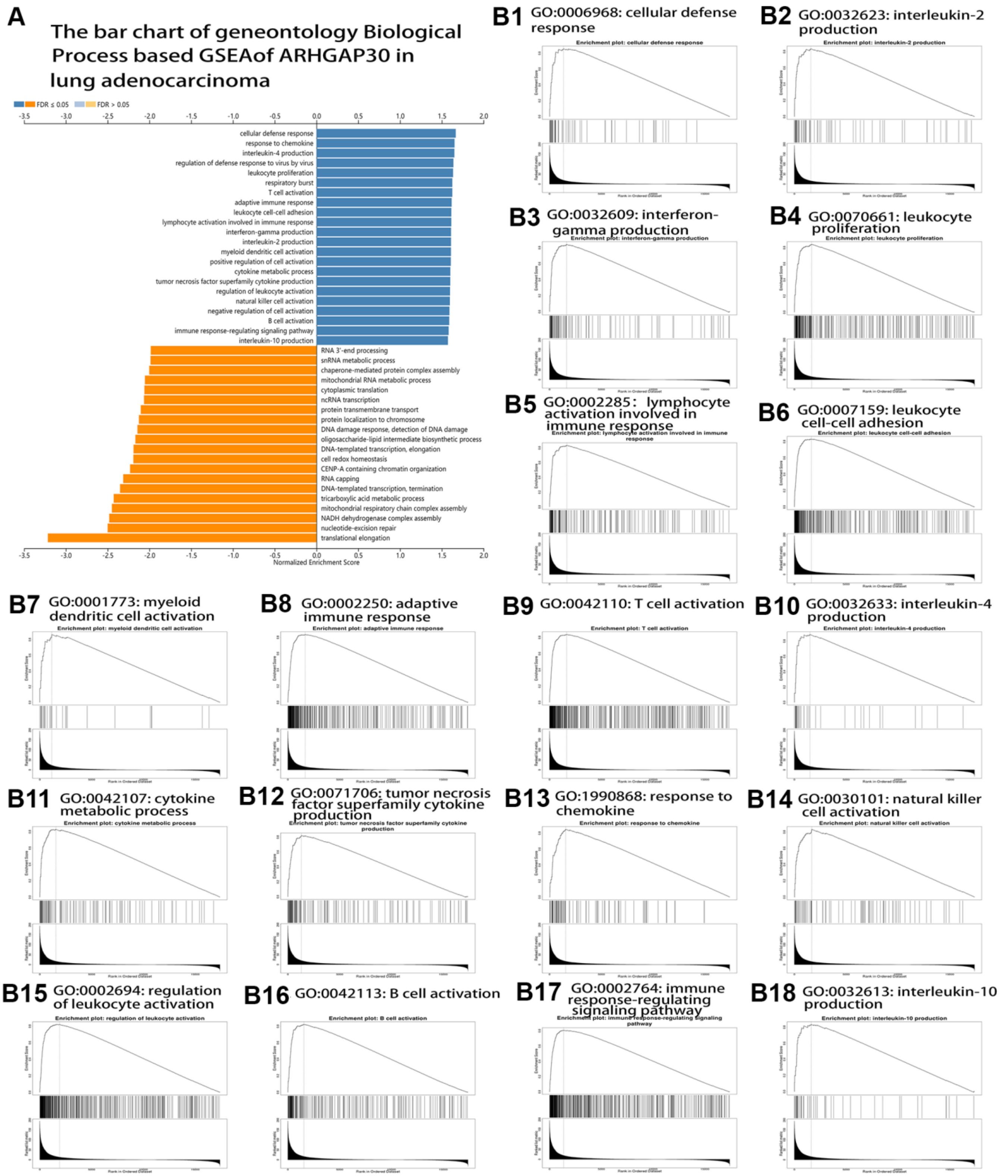


**Supplementary Figure 4. Gene ontology biological process-based GSEA of *ARHGAP30* in lung adenocarcinoma (LUAD).** (**A**) Bar chart of Gene Ontology Biological Process-based GSEA of *ARHGAP30* in LUAD (FDR < 0.05). (**B1**–**B18**) GSEA enrichment analysis plots of 18 tumors immune-related Gene Ontology Biological Process gene sets (FDR < 0.05).

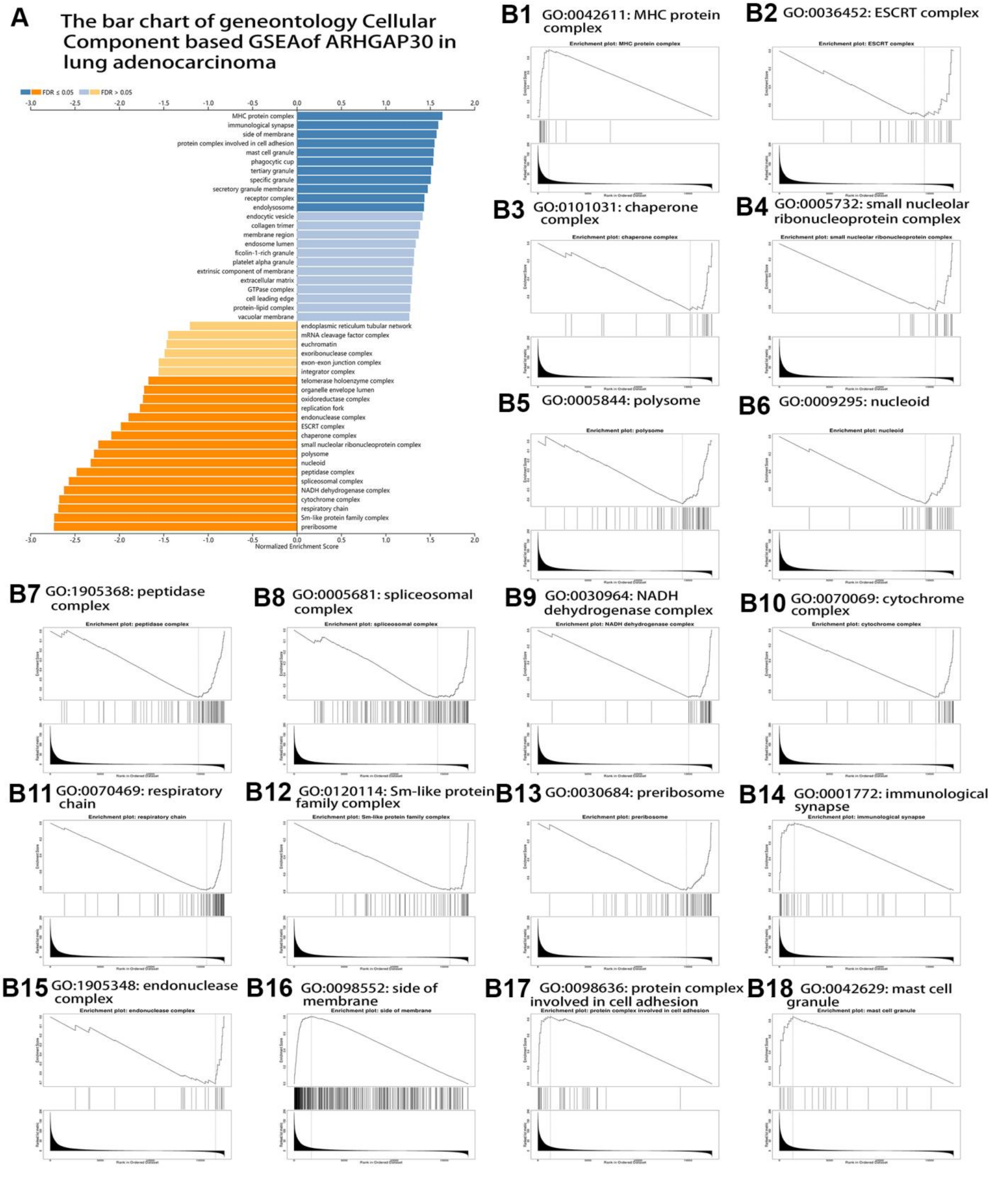


**Supplementary Figure 5. Gene ontology cellular component-based GSEA of *ARHGAP30* in lung adenocarcinoma (LUAD).** (**A**) Bar chart of Gene Ontology Cellular Component-based GSEA of *ARHGAP30* in LUAD (FDR < 0.05). (**B1**–**B18**) GSEA enrichment analysis plots of 18 tumors immune-related Gene Ontology Cellular Component gene sets (FDR < 0.05).

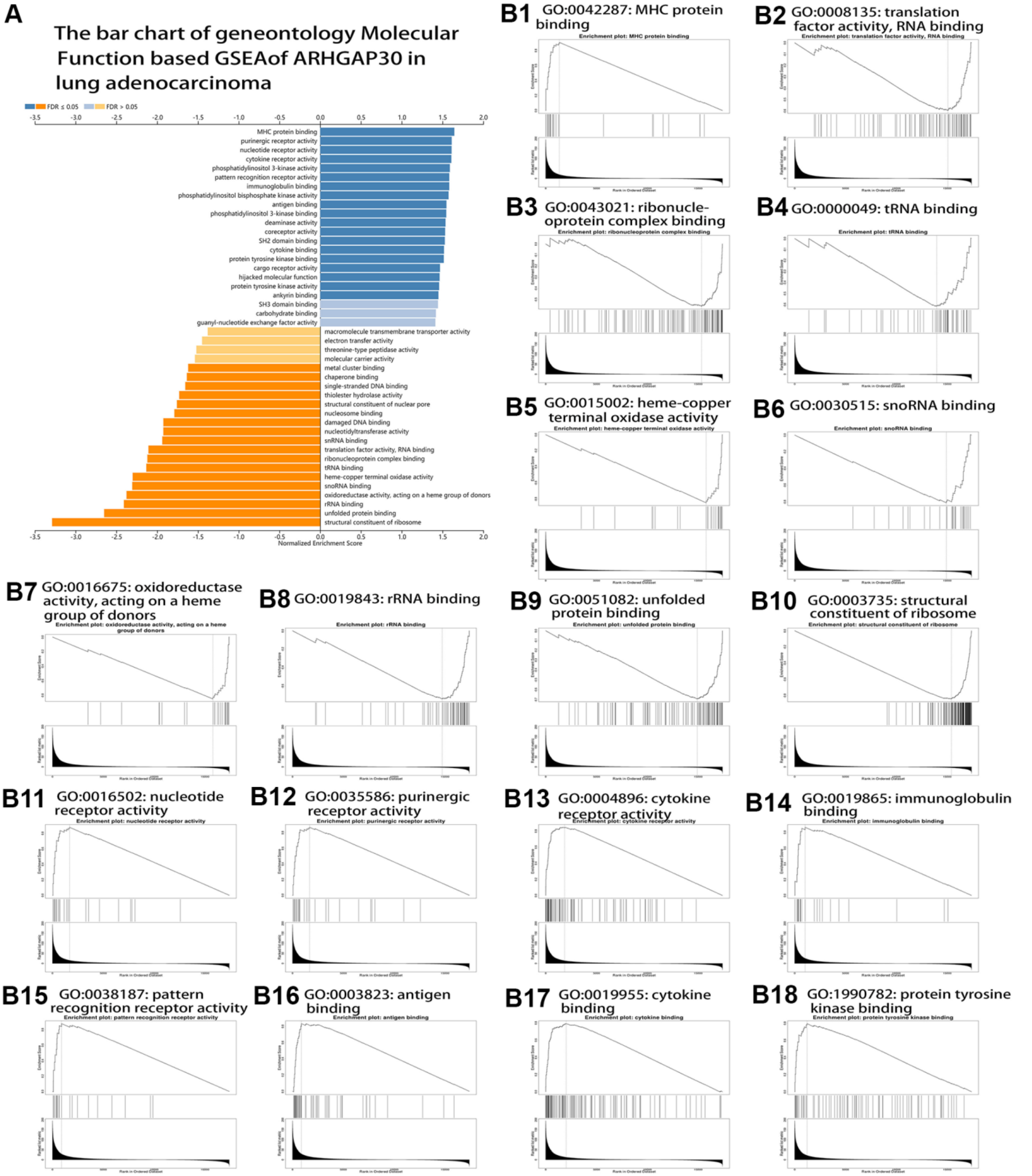


**Supplementary Figure 6. Gene ontology molecular function-based GSEA of *ARHGAP30* in lung adenocarcinoma (LUAD).** (**A**) Bar chart of Gene Ontology Molecular Function-based GSEA of *ARHGAP30* in LUAD (FDR < 0.05). (**B1**–**B18**) GSEA enrichment analysis plots of 18 tumors immune-related Gene Ontology Molecular Function gene sets (FDR < 0.05).

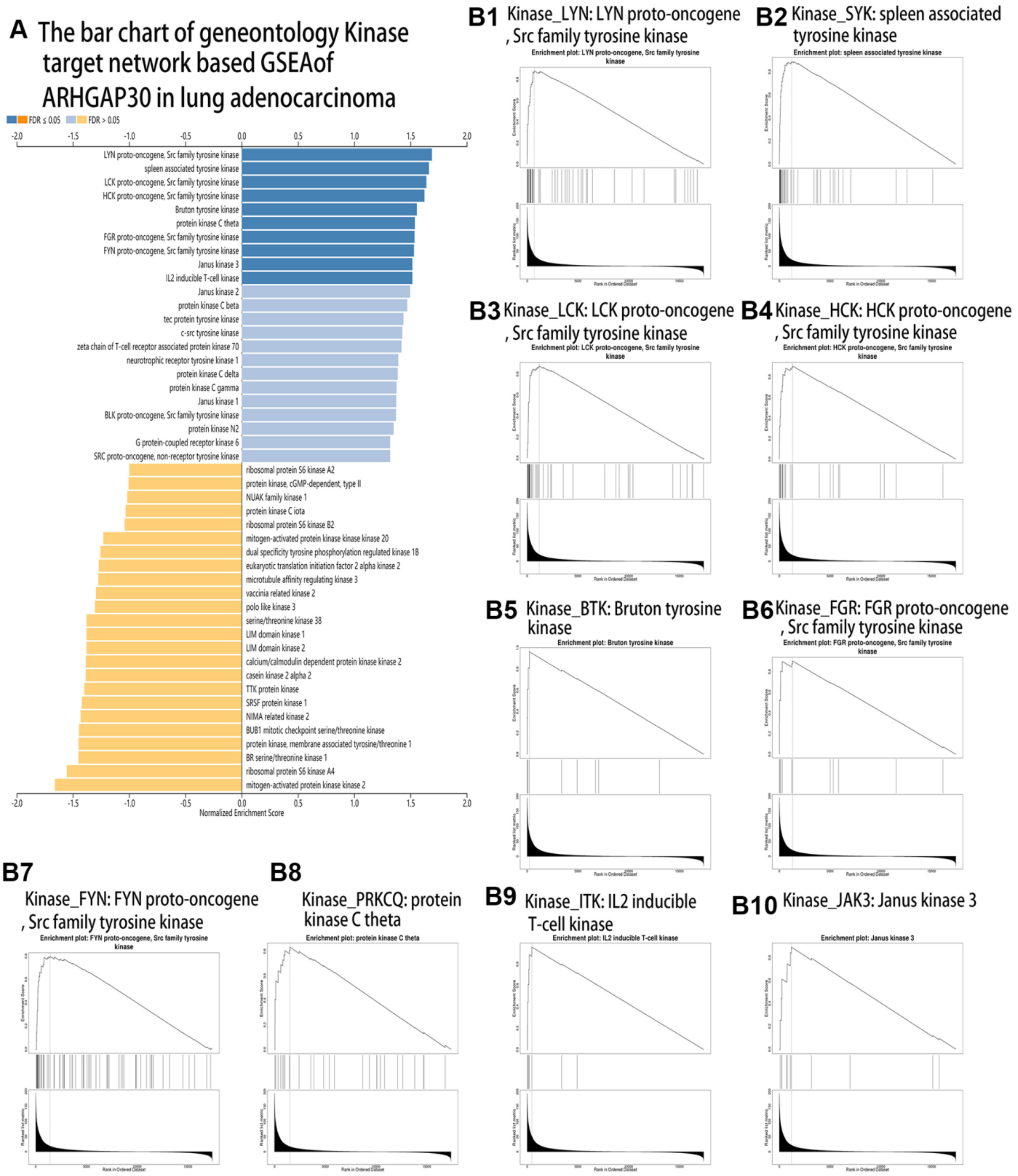


**Supplementary Figure 7. Kinase target network-based GSEA of *ARHGAP30* in lung adenocarcinoma (LUAD).** (**A**) Bar chart of Kinase Target Network-based GSEA of *ARHGAP30* in LUAD (FDR < 0.05). (**B1**–**B10**) GSEA enrichment analysis plots of 10 tumor immune-related Kinase Target Network gene sets (FDR < 0.05).

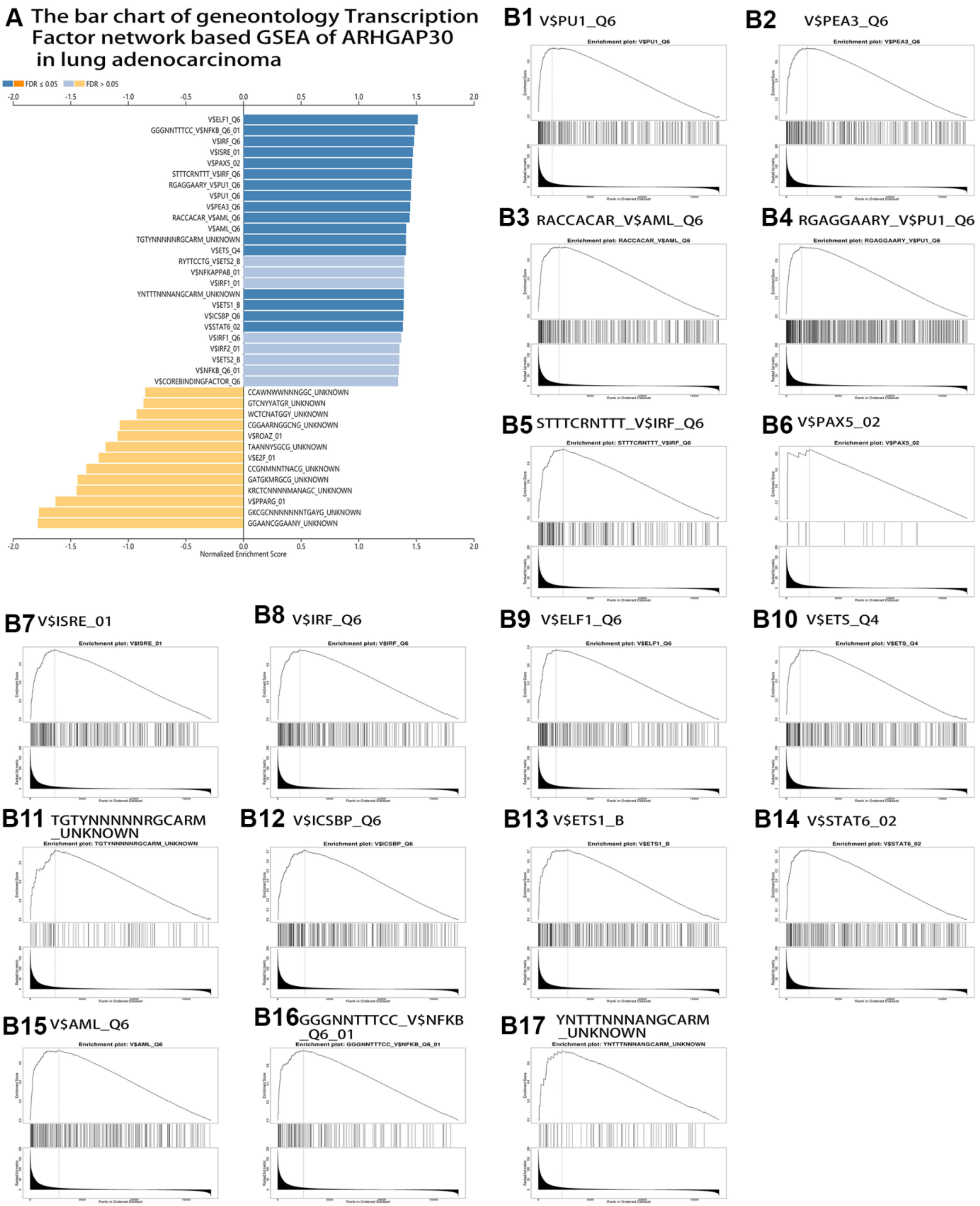


**Supplementary Figure 8. Transcription factor network-based GSEA of *ARHGAP30* in lung adenocarcinoma (LUAD).** (**A**) Bar chart of Transcription Factor Network-based GSEA of *ARHGAP30* in LUAD (FDR < 0.05). (**B1**–**B17**) GSEA enrichment analysis plots of 17 tumor immune-related Transcription Factor Network gene sets (FDR < 0.05).

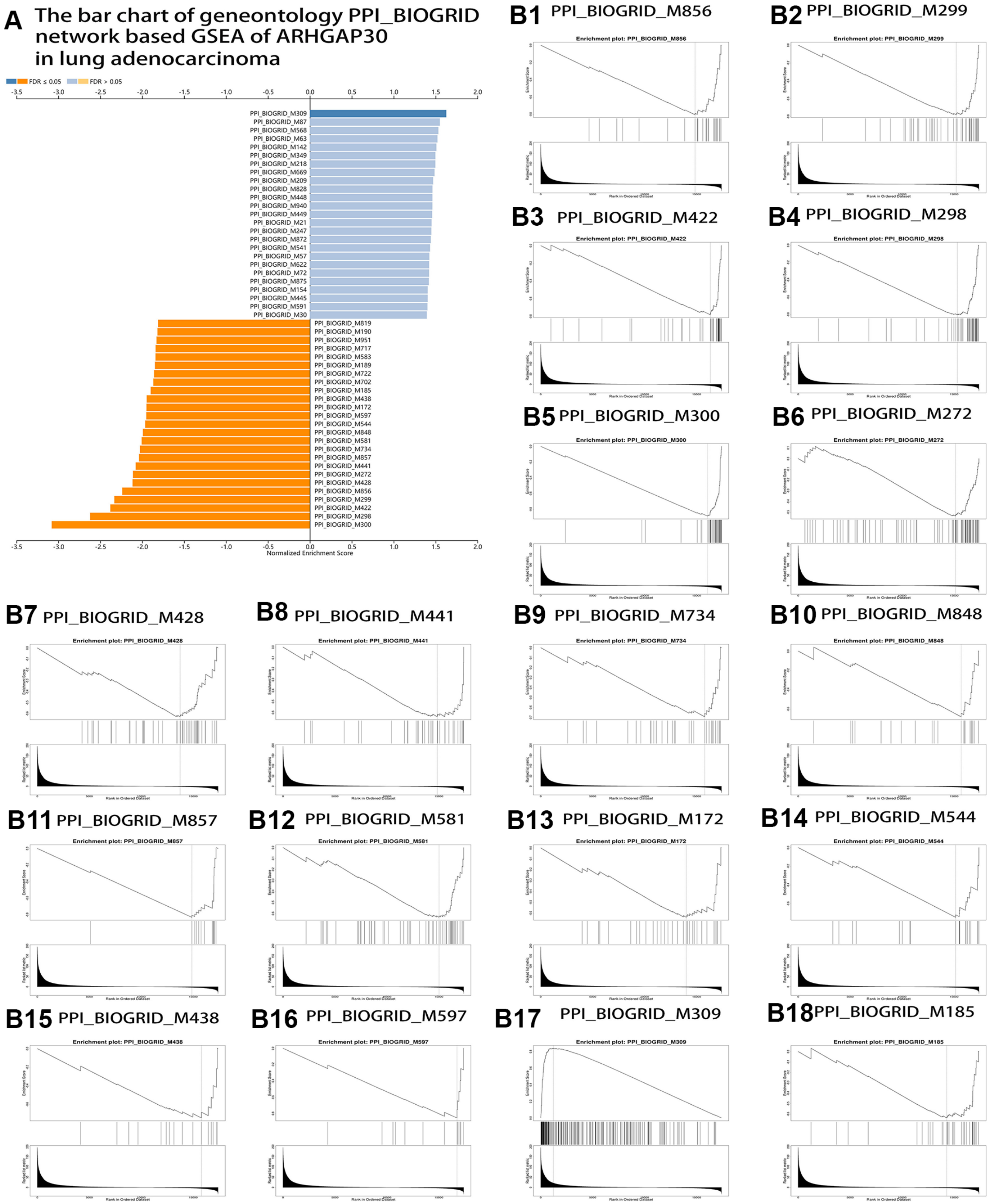


**Supplementary Figure 9. PPI BIOGRID network-based GSEA of *ARHGAP30* in lung adenocarcinoma (LUAD).** (**A**) Bar chart of PPI BIOGRID Network-based GSEA of *ARHGAP30* in LUAD (FDR < 0.05). (**B1–B18**) GSEA enrichment analysis plots of 18 tumor immune-related PPI BIOGRID Network gene sets (FDR < 0.05).

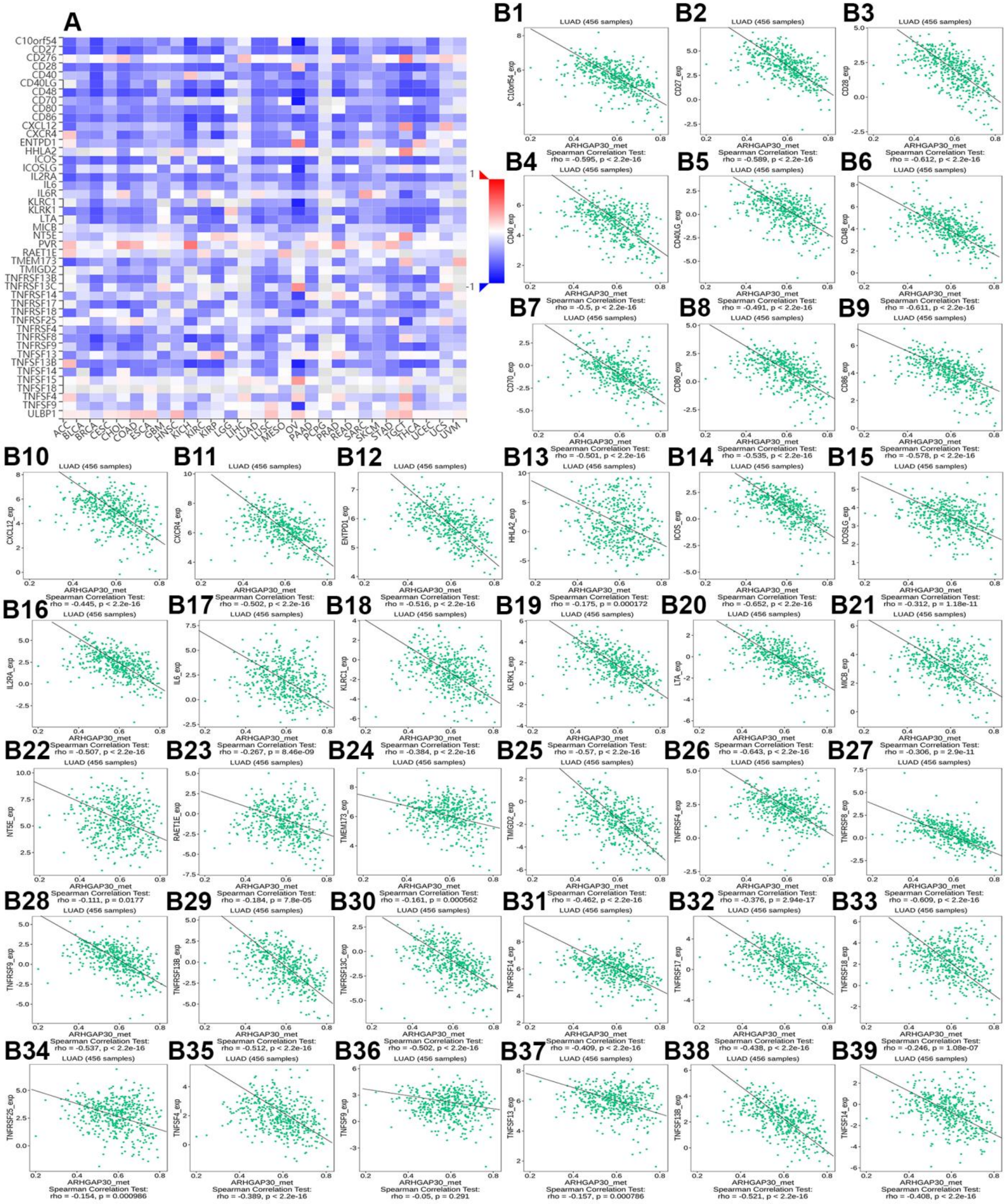


**Supplementary Figure 10. The correlation between the expression of *ARHGAP30* and MHC molecules.** (**A**) Heat map of Spearman correlations between *ARHGAP30* expression and MHC molecules across human cancers. (**B1**–**B39**) Scatter plots showing the positive correlation between *ARHGAP30* expression and MHC molecules in the treatment of lung adenocarcinoma.

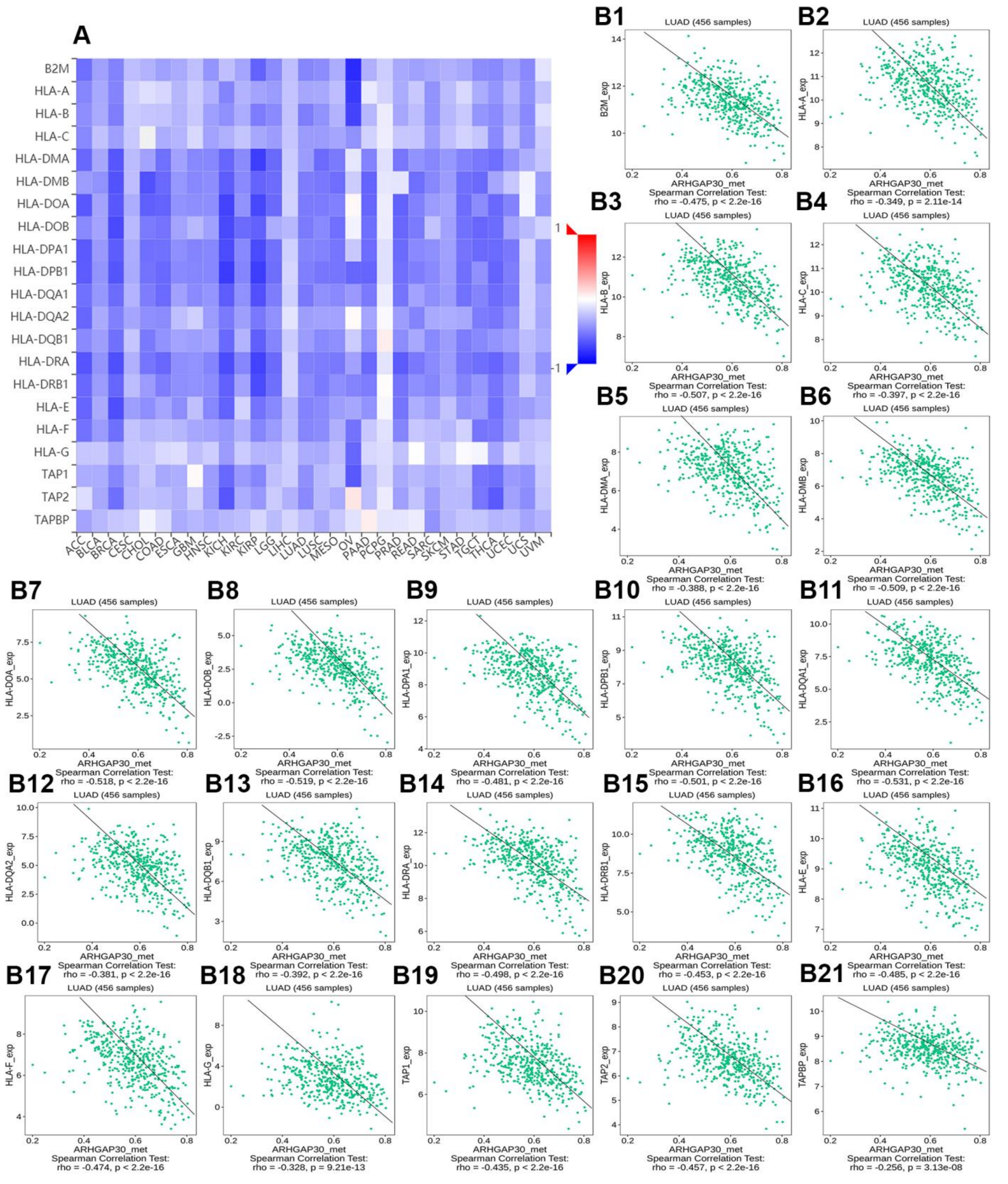


**Supplementary Figure 11. The correlation between the DNA methylation of *ARHGAP30* and MHC molecules.** (**A**) Heat map of Spearman correlations between DNA methylation of *ARHGAP30* and MHC molecules across human cancers. (**B1**–**B21**) Scatter plots showing the negative correlation between DNA methylation of *ARHGAP30* and MHC molecules in the treatment of lung adenocarcinoma.

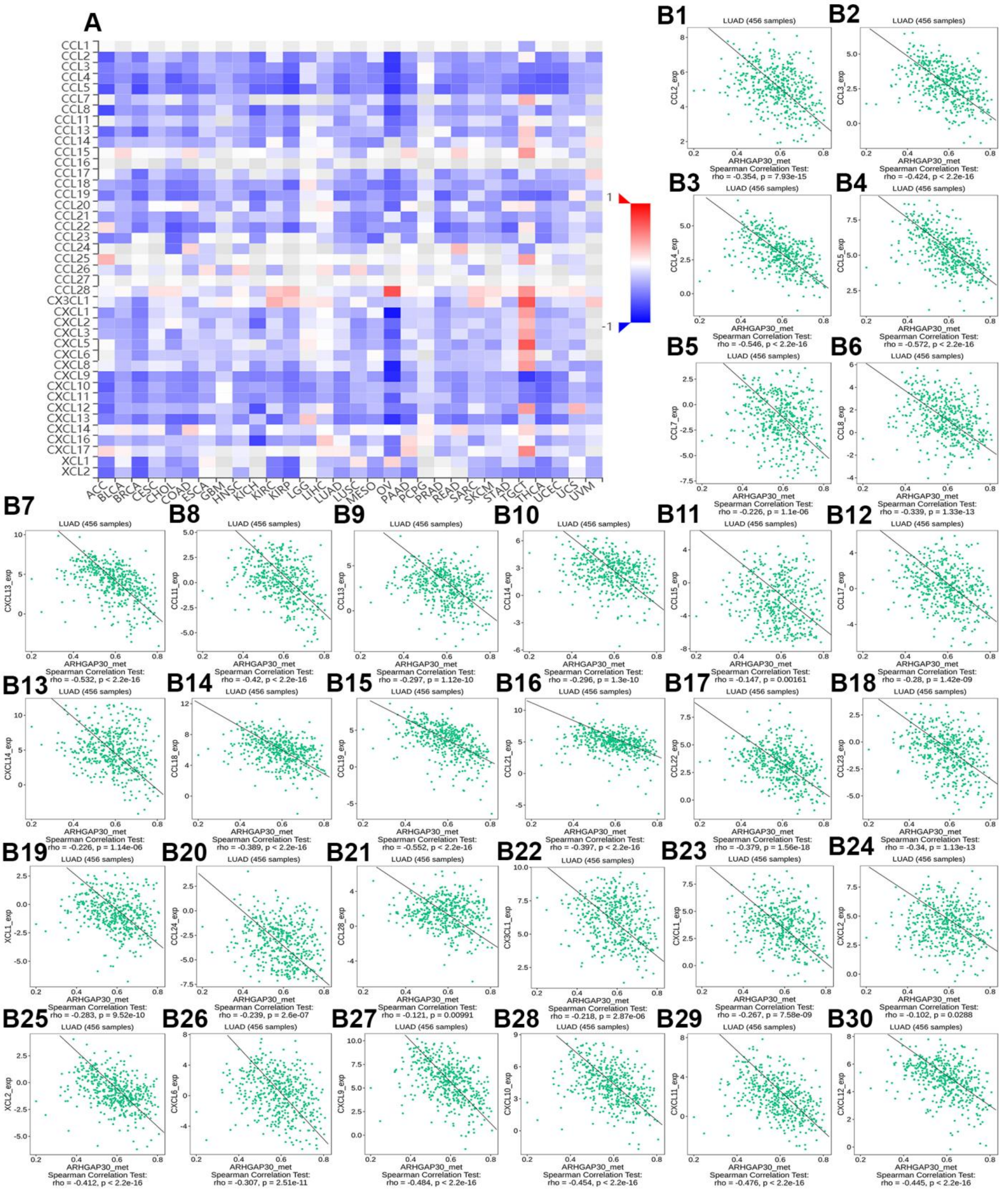


**Supplementary Figure 12. The correlation between the expression of *ARHGAP30* and chemokines.** (**A**) Heat map of Spearman correlations between *ARHGAP30* expression and chemokines across human cancers. (**B1**–**B30**) Scatter plots showing the positive correlation between *ARHGAP30* expression and chemokines in the treatment of lung adenocarcinoma.

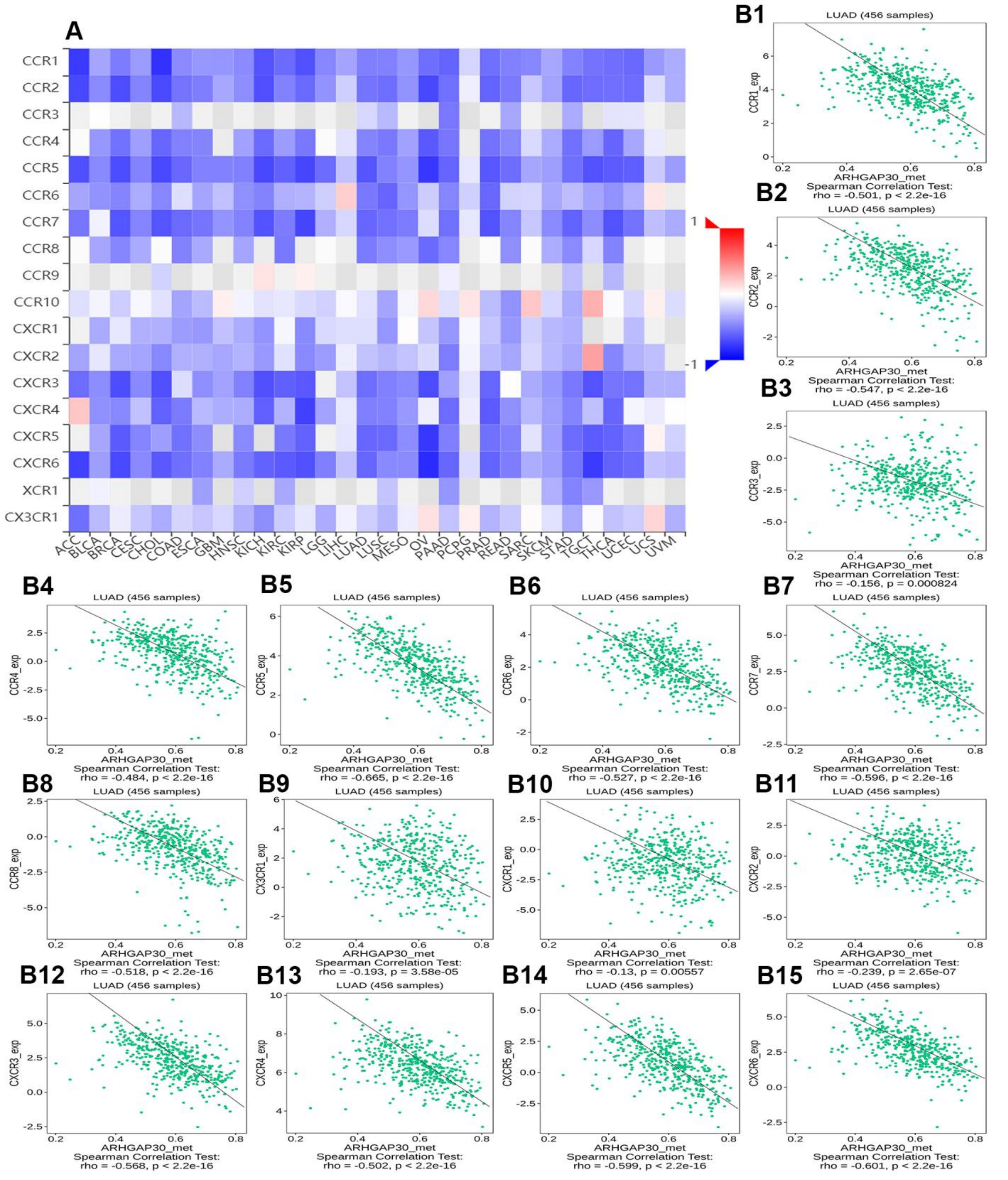


**Supplementary Figure 13. The correlation between the expression of *ARHGAP30* and Chemokine Receptors.** (**A**) Heat map of Spearman correlations between *ARHGAP30* expression and chemokine receptors across human cancers. (**B1**–**B15**) Scatter plots showing the positive correlation between *ARHGAP30* expression and chemokine receptors in the treatment of lung adenocarcinoma.
