# Supplementary material for: DNA methylation of ARHGAP30 is negatively associated with ARHGAP30 expression in lung adenocarcinoma, which reduces tumor immunity and is detrimental to patient survival
Source: Aging (Albany NY). 2021 Dec 15;13(24):25799–845. doi: 10.18632/aging.203762 (PMC8751594; doi:10.18632/aging.203762)
Supplement: Supplementary Tables [file aging-13-203762-s002.pdf]

## SUPPLEMENTARY TABLES

**Supplementary Table 1. Expression of *ARHGAP30* mRNA in different subgroups.**

| Comparison | Statistical significance | Comparison | Statistical significance |
|---|---|---|---|
| **Sample types** | | Age(21-40Yrs)-vs-Age(81-100Yrs) | 2.99E-01 |
| Normal-vs-Primary | 1.62E-12 | Age(41-60Yrs)-vs-Age(61-80Yrs) | 2.83E-01 |
| **Individual cancer stages** | | Age(41-60Yrs)-vs-Age(81-100Yrs) | 6.00E-02 |
| Normal-vs-Stage1 | 1.11E-16 | Age(61-80Yrs)-vs-Age(81-100Yrs) | 1.18E-01 |
| Normal-vs-Stage2 | 1.11E-16 | **Patient's smoking habit** | |
| Normal-vs-Stage3 | 1.62E-12 | Normal-vs-Non smoker | 1.34E-11 |
| Normal-vs-Stage4 | 1.66E-13 | Normal-vs-Smoker | <1E-12 |
| Stage1-vs-Stage2 | 2.62E-01 | Normal-vs-Reformed smoker1 | 1.62E-12 |
| Stage1-vs-Stage3 | 2.19E-04 | Normal-vs-Reformed smoker2 | <1E-12 |
| Stage1-vs-Stage4 | 1.71E-02 | Non smoker-vs-Smoker | 2.04E-01 |
| Stage2-vs-Stage3 | 3.79E-02 | Non smoker-vs-Reformed smoker1 | 4.57E-01 |
| Stage2-vs-Stage4 | 1.30E-01 | Non smoker-vs-Reformed smoker2 | 2.59E-01 |
| Stage3-vs-Stage4 | 9.91E-01 | Smoker-vs-Reformed smoker1 | 5.14E-01 |
| **Patient's race** | | Smoker-vs-Reformed smoker2 | 6.98E-01 |
| Normal-vs-Caucasian | 1.62E-12 | Reformed smoker1-vs-Reformed smoker2 | 7.22E-01 |
| Normal-vs-African American | 1.75E-12 | **Nodal metastasis status** | |
| Normal-vs-Asian | 9.72E-07 | Normal-vs-N0 | 1.62E-12 |
| Caucasian-vs-African American | 3.96E-01 | Normal-vs-N1 | <1E-12 |
| Caucasian-vs-Asian | 1.38E-01 | Normal-vs-N2 | <1E-12 |
| African American-vs-Asian | 2.29E-01 | Normal-vs-N3 | 5.83E-02 |
| **Patient's gender** | | N0-vs-N1 | 1.20E-01 |
| Normal-vs-Male | 1.11E-16 | N0-vs-N2 | 1.28E-03 |
| Normal-vs-Female | <1E-12 | N0-vs-N3 | 9.73E-01 |
| Male-vs-Female | 1.78E-02 | N1-vs-N2 | 2.39E-01 |
| **Patient's age** | | N1-vs-N3 | 8.20E-01 |
| Normal-vs-Age(21-40Yrs) | 1.54E-03 | N2-vs-N3 | 5.56E-01 |
| Normal-vs-Age(41-60Yrs) | 1.62E-12 | **TP53 mutation status** | |
| Normal-vs-Age(61-80Yrs) | <1E-12 | Normal-vs-TP53-Mutant | 1.62E-12 |
| Normal-vs-Age(81-100Yrs) | 1.59E-04 | Normal-vs-TP53-NonMutant | <1E-12 |
| Age(21-40Yrs)-vs-Age(41-60Yrs) | 6.98E-01 | TP53-Mutant-vs-TP53-NonMutant | 9.66E-01 |
| Age(21-40Yrs)-vs-Age(61-80Yrs) | 5.40E-01 | | |

**Supplementary Table 2. Expression of different subgroups of *ARHGAP30* protein.**

| Comparison | Statistical significance | Comparison | Statistical significance |
|---|---|---|---|
| **Sample types** | | Age(41-60Yrs)-vs-Age(61-80Yrs) | 6.09E-03 |
| Normal-vs-Primary | 2.03E-08 | Age(41-60Yrs)-vs-Age(81-100Yrs) | 3.89E-01 |
| **Individual cancer stages** | | Age(61-80Yrs)-vs-Age(81-100Yrs) | 6.12E-01 |
| Normal-vs-Stage1 | 9.45E-05 | **Patient's weight** | |
| Normal-vs-Stage2 | 4.59E-05 | Normal-vs-Normal Weight | 3.09E-07 |
| Normal-vs-Stage3 | 1.49E-02 | Normal-vs-Extreme Weight | 6.86E-02 |
| Normal-vs-Stage4 | NA | Normal-vs-Obese | 4.74E-02 |
| Stage1-vs-Stage2 | 1.95E-01 | Normal-vs-Extreme Obese | 5.93E-01 |
| Stage1-vs-Stage3 | 9.76E-01 | Normal Weight-vs-Extreme Weight | 6.60E-02 |
| Stage1-vs-Stage4 | NA | Normal Weight-vs-Obese | 6.49E-01 |
| Stage2-vs-Stage3 | 2.94E-01 | Normal Weight-vs-Extreme Obese | 8.59E-01 |
| Stage2-vs-Stage4 | NA | Extreme Weight-vs-Obese | 4.18E-01 |
| Stage3-vs-Stage4 | NA | Extreme Weight-vs-Extreme Obese | 8.34E-01 |
| **Patient's race** | | Obese-vs-Extreme Obese | 9.64E-01 |
| Normal-vs-Caucasian | 3.61E-02 | **Tumor's grade** | |
| Normal-vs-African American | NA | Normal-vs-Grade1 | 6.90E-03 |
| Normal-vs-Asian | NA | Normal-vs-Grade2 | 6.10E-05 |
| Caucasian-vs-African American | NA | Normal-vs-Grade3 | 5.87E-04 |
| Caucasian-vs-Asian | NA | Grade1-vs-Grade2 | 2.75E-01 |
| African American-vs-Asian | NA | Grade1-vs-Grade3 | 6.04E-01 |
| **Patient's gender** | | Grade2-vs-Grade3 | <1E-12 |
| Normal-vs-Male | 2.68E-05 | **Tumor histology** | |
| Normal-vs-Female | 3.91E-06 | Normal-vs-Lepidic adenocarcinoma | 1.99E-01 |
| Male-vs-Female | 2.29E-01 | Normal-vs-Papillary adenocarcinoma | 9.59E-02 |
| **Patient's age** | | Normal-vs-Squamous cell carcinoma | NA |
| Normal-vs-Age(21-40Yrs) | 9.32E-02 | Normal-vs-Adenocarcinoma | 5.52E-04 |
| Normal-vs-Age(41-60Yrs) | 9.87E-08 | Normal-vs-Colloid adenocarcinoma | NA |
| Normal-vs-Age(61-80Yrs) | 1.25E-03 | Normal-vs-Acinar adenocarcinoma | 1.58E-03 |
| Normal-vs-Age(81-100Yrs) | 9.39E-01 | Normal-vs-Solid adenocarcinoma | 6.70E-02 |
| Age(21-40Yrs)-vs-Age(41-60Yrs) | 6.06E-01 | Normal-vs-Other | 2.70E-03 |
| Age(21-40Yrs)-vs-Age(61-80Yrs) | 1.93E-01 | Papillary adenocarcinoma-vs-Other | 4.01E-01 |
| Age(21-40Yrs)-vs-Age(81-100Yrs) | 2.93E-01 | Adenocarcinoma-vs-Other | 2.56E-01 |
